# Early Hidden-Crack Detection in Circular Magnetic Encoder Rings Through FM-AM Signal Decoupling

**DOI:** 10.3390/s26165135

**Published:** 2026-08-14

**Authors:** Hai Xu, Bin Wang, Zhenyang Wu, Jinhua Guan, Dangwei Guo, Xiaolong Fan

**Affiliations:** 1 National Key Laboratory of Particle Transport and Separation Technology, Tianjin 300180, China; 2 Institute of Physical and Chemical Engineering of Nuclear Industry, 168 Jintang Road, Tianjin 300180, China; 3 Key Laboratory for Magnetism and Magnetic Materials of the Ministry of Education, Lanzhou University, 222 Tianshui South Road, Lanzhou 730000, China

**Keywords:** magnetic encoder, hidden crack detection, tunnel magnetoresistance sensor, Hilbert transform, amplitude-modulated–frequency-modulated demodulation, condition monitoring

## Abstract

Early hidden cracks in magnetic encoder rings induce only slight magnetic perturbations at the incipient stage, yet they may evolve into missing-pole, demagnetization, or severe waveform-distortion faults that degrade angular-displacement measurement and closed-loop control. This study establishes a physical mapping between crack-induced magnetization nonuniformity, pole-pitch deviation, and the amplitude-modulated and frequency-modulated components of the measured magnetic signal. A precision-controlled experimental platform equipped with a self-developed tunnel magnetoresistance read head, a precision rotary stage, multi-axis positioning stages, and laser displacement sensing was built to measure the spatial magnetic field during shaft rotation. Fast Fourier transform-based band-pass filtering, Hilbert transform-based demodulation, and sixth-order Butterworth low-pass filtering were used to extract the instantaneous amplitude $A_{i}$ and instantaneous angular frequency $\omega_{i}$; coefficient-of-variation analysis was then used to construct the diagnostic indicators. Experiments on intact, hidden-crack, and visible-crack states show that the CV of $A_{i}$ and the CV of $\omega_{i}$ sensitively capture weak crack-related fluctuations and increase monotonically with damage severity. The method does not require a high-accuracy external reference and shows promise for online monitoring of magnetic encoder rings and related electromagnetic sensing elements.

## 1. Introduction

High-accuracy angular-displacement measurement is a prerequisite for precision motion control in machine-tool spindles, servo drives, industrial robots, and other high-end rotating equipment [1,2]. Compared with optical encoders, magnetic position sensors and magnetic encoders are more tolerant to dust, oil mist, vibration, and other harsh industrial conditions while retaining compact size, contactless operation, and favorable cost-performance characteristics [3,4]. Recent advances in magnetic encoder technology have further expanded their applicability in compact industrial motion-control systems [5].

Despite these advantages, the measurement performance of magnetic encoder rings remains strongly dependent on the health of the magnetized pole array and the read-head signal quality. Manufacturing defects, local damage, assembly errors, and environmental disturbances can all distort the encoded field distribution and thereby degrade the angular output [6,7]. Among these fault modes, hidden cracks are particularly challenging because the initial damage is extremely weak and is often invisible to the naked eye or to simple magnetic viewing methods. If undetected, such defects may progressively develop into more severe missing-pole or demagnetized regions, eventually threatening control accuracy and operational reliability.

For precision equipment, the practical difficulty is not only whether a severe defect can be detected after the output has already deteriorated, but also whether early degradation can be recognized before it produces a clear measurement failure. A magnetic encoder ring contains a periodically magnetized pole pattern, so local damage does not necessarily appear as a large isolated anomaly in the raw waveform. Instead, it may be distributed over the carrier and its modulation sidebands, where it is easily masked by eccentricity, sensor lift-off distance variation, vibration, and electronic noise. In particular, radial eccentricity and pole-wheel form errors can introduce non-negligible magnetic-field distortions in side-of-shaft rotary magnetic encoders [8]. This feature makes hidden-crack diagnosis different from ordinary waveform-threshold detection: the key information is embedded in small changes in the regularity of the modulated magnetic signal.

Existing studies on magnetic encoders have mainly focused on structural design, error-source analysis, and algorithmic compensation of explicit nonlinearities [9,10]. These approaches are valuable for improving measurement performance, but they are generally more sensitive to obvious faults or macroscopic error sources than to subtle early-stage degradation. Related work on encoder-signal-based condition monitoring has shown that weak fluctuations embedded in position signals can reveal mechanical health conditions, provided that suitable signal decomposition and denoising methods are available [11,12].

More general data-driven fault-diagnosis frameworks, including vibration-based structural health monitoring, digital-twin-assisted diagnosis, and contrastive or self-supervised learning methods, are powerful for multi-source and cross-domain fault classification [13,14,15,16]. The present study is complementary to these approaches because it focuses on a specific physical mechanism in magnetic encoder rings and converts hidden-crack-induced pole degradation into interpretable AM–FM signal descriptors.

In this study, hidden-crack diagnosis is reformulated as an AM–FM signal-decoupling problem. The central idea is that hidden cracks change both the magnetization strength of local poles and the effective spacing between adjacent poles. These two effects respectively manifest as instantaneous-amplitude nonuniformity and frequency fluctuation in the acquired magnetic waveform. On this basis, the main contributions of this work are as follows:

1. A physical mapping is established between hidden-crack-induced pole degradation and the AM/FM components of the measured magnetic signal, allowing the magnetic encoder signal to be modeled as a compound frequency modulation–amplitude modulation (FM–AM) waveform.
2. A precision-controlled experimental platform based on a self-developed tunnel magnetoresistance (TMR) read head and precision alignment units is constructed to minimize eccentricity-related interference and interference related to sensor lift-off distance during controlled data acquisition.
3. A feature-extraction strategy based on band-pass filtering, Hilbert transform-based demodulation, secondary low-pass filtering, and coefficient-of-variation (CV) values is proposed to quantify magnetization uniformity and pole-spacing consistency under fixed and comparable measurement conditions without using a high-accuracy external reference.

## 2. Materials and Methods

### 2.1. Physical Mapping and Signal Model

A hidden crack produces two coupled effects in a magnetic encoder ring. Local material damage changes the magnetization intensity of the affected pole region, which appears as instantaneous-amplitude nonuniformity in the magnetic signal acquired by the read head. Crack-induced geometric distortion or magnetic-field redistribution also changes the effective spacing and field transition between adjacent poles, which appears as instantaneous-frequency fluctuation. This interpretation is consistent with magnetic-wheel-encoder health studies showing that local degradation can appear as subtle waveform and spectral changes [17]. Under fixed-axis rotation, the measured magnetic flux density can therefore be represented by a compound FM–AM model

(1) $$B\left(t\right)=A_{i}\left(t\right)\cos\;\left[\int_{0}^{t}\omega_{i}\left(\tau \right)\;d\tau +\theta_{0}\right].$$

where $A_{i}\left(t\right)$ is the instantaneous amplitude mainly associated with pole-magnetization uniformity, $\omega_{i}\left(t\right)$ is the instantaneous angular frequency mainly associated with pole-passage regularity, and $\theta_{0}$ is the initial phase. In this model, a crack-related weakening or redistribution of local magnetization increases the dispersion of $A_{i}\left(t\right)$, whereas a crack-related change in effective pole spacing or field transition increases the fluctuation of $\omega_{i}\left(t\right)$. The model is not a full electromagnetic field simulation; rather, it provides a compact diagnostic representation that links the measured waveform to the two physical degradation pathways required by the proposed indicators.

### 2.2. Experimental Platform

The main crack-severity experiment was intentionally conducted under controlled and repeatable conditions. This idealized setting was used to highlight the physical relationship between crack-induced pole degradation and AM–FM feature changes before discussing non-ideal operating factors. The method assumes fixed-axis rotation, a fixed read-head position during each acquisition, a known and stable pole number, an identifiable pole-passage carrier in the measured spectrum, and comparable installation conditions among the damage states.

**Figure 1:**
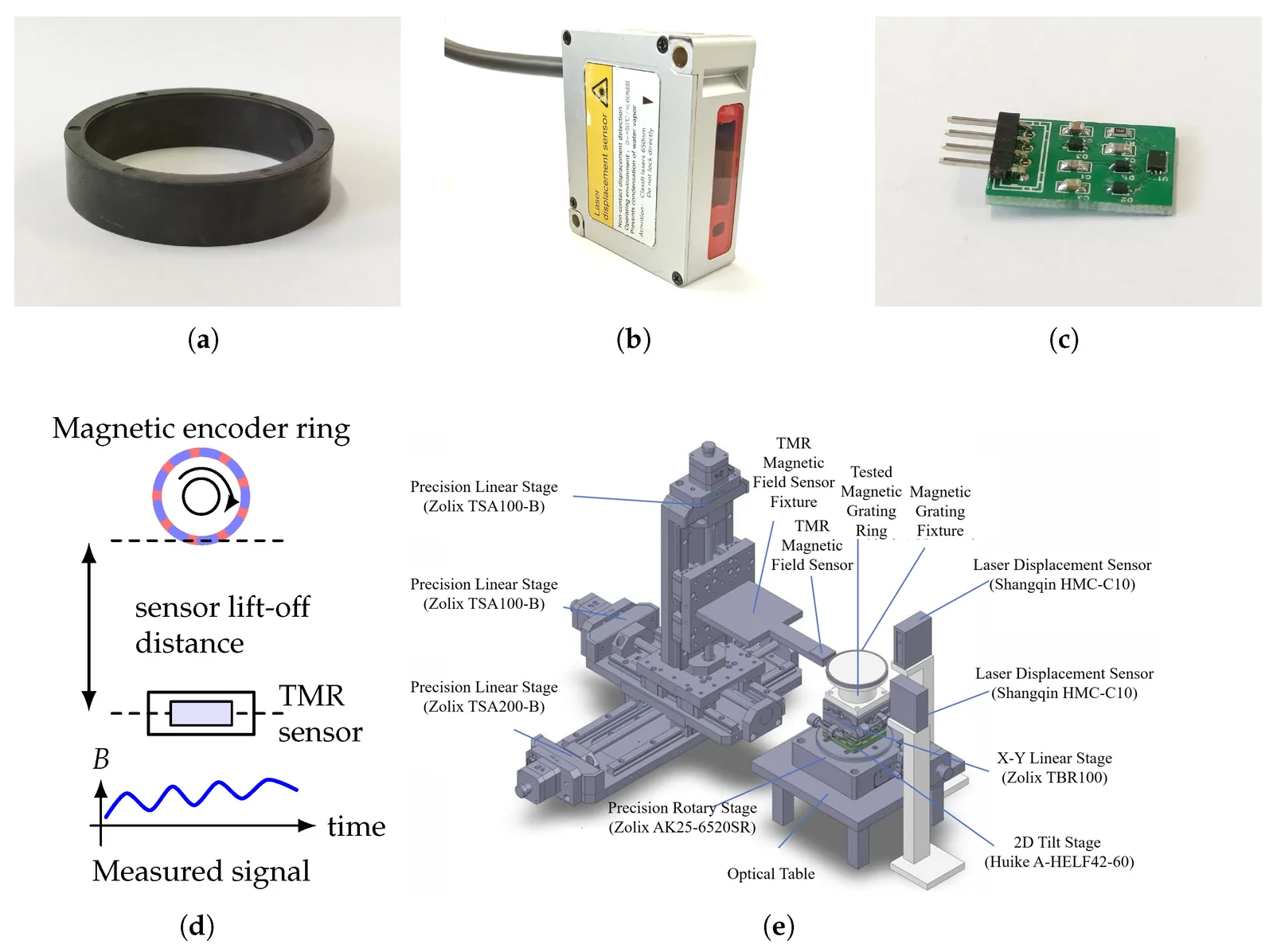
Experimental platform for magnetic-signal acquisition from a magnetic encoder ring. (**a**) Tested magnetic encoder ring. (**b**) Laser displacement sensor used for alignment and radial-displacement monitoring. (**c**) Self-developed TMR read head. (**d**) Definition of the sensor lift-off distance between the ring surface and the TMR sensing surface, together with the acquired magnetic signal. (**e**) Schematic layout of the rotary stage, positioning stages, read head, and displacement sensor.

To reproduce the working state of a magnetic encoder ring under fixed-axis rotation, a precision-controlled measurement platform was assembled, as shown schematically in Figure 1. The tested specimen was an MR5232 commercial pre-magnetized multipole magnetic encoder ring manufactured by Jiangsu Aoming Technology (Yangzhou, China). The model information and nominal dimensions were taken from manufacturer-supplied product information: inner diameter 43±0.03 mm, outer diameter 50.9±0.03 mm, width 12±0.1 mm, 32 magnetic poles, and nominal pole width 5 mm. The detailed material formulation and industrial magnetizing-fixture parameters were not provided by the manufacturer and were therefore not treated as variables in this study. The encoder ring was driven by a Zolix AK25-6520SR precision rotary stage (Zolix Instruments Co., Ltd., Beijing, China) with a positioning accuracy of ±1 arcsec and a repeatability better than 0.5 arcsec. A Zolix TBR100 two-dimensional translation stage (Zolix Instruments Co., Ltd., Beijing, China) was installed beneath the rotary stage to align the magnetic encoder ring center with the rotary axis. An HMC-C10 laser displacement sensor was used during alignment to monitor residual offset.

The radial displacement of the magnetic encoder ring outer surface was monitored during rotation. This runout-related signal includes mounting eccentricity, rotary-axis error, and possible roundness or form error of the ring. The ring center was adjusted using the two-dimensional translation stage, with the laser displacement sensor used as the alignment feedback, to reduce the dominant first-order eccentric component before magnetic-signal acquisition. The remaining runout and form-error effects were treated as controlled geometric conditions because the same installation, rotation condition, sampling interval, read-head position, and processing workflow were maintained for the intact, hidden-crack, and visible-crack comparisons.

The magnetic signal was acquired by a self-developed TMR read head based on a MultiDimension TMR2652D linear TMR chip (MultiDimension Technology Co., Ltd., Suzhou, China). In the present setup, the read head was mounted on a three-axis positioning unit composed of one TSA200-B stage for the *x* direction and two TSA100-B stages for the *y* and *z* directions (Zolix Instruments Co., Ltd., Beijing, China), enabling fine control of the sensor position and sensor lift-off distance. A Python-based acquisition program (Python 3.10) coordinated the rotary stage, TMR read head, and laser displacement sensor to achieve synchronized and automated acquisition of magnetic, displacement, and angular-position signals. The data-acquisition interval was fixed at 100 ms, corresponding to a sampling rate of 10 Hz.

All magnetic signals were acquired by uniform time sampling rather than angular sampling. The rotary speed was set to 0.625 rpm, corresponding to a rotation period of 96 s. Each raw acquisition lasted 750 s and was truncated to seven complete revolutions before feature extraction, giving an effective duration of 672 s and approximately 6720 data points. For the 32-pole encoder ring, this corresponds to approximately 960 data points per revolution and 30 data points per pole.

Before each measurement, the encoder ring was adjusted so that the residual radial offset was minimized according to the laser displacement reading. The sensor lift-off distance is defined here as the normal air-gap distance between the sensitive surface of the TMR read head and the outer surface of the encoder ring. This distance was set by the *z*-axis positioning stage and kept unchanged during each individual acquisition. Except for the intentionally changed damage state or sensor lift-off distance, the mechanical rotation condition, sampling interval, read-head position, and data-processing program were kept identical among the crack-state comparisons.

### 2.3. Specimen Preparation and Damage-State Design

Four damage states were prepared sequentially on the same magnetic encoder ring: intact, hidden crack, visible 1 mm crack, and visible 2 mm crack. The intact state was first measured without intentional damage and was used as the baseline. The hidden-crack state was produced by localized mechanical impact in a selected region of the magnetic ring. The impact induced internal cracking, after which the ring was restored so that no externally open crack remained. The hidden crack is therefore described by its preparation method and inspection invisibility rather than by a directly measured crack size. The visible-crack states were then prepared by inserting fillers inside the ring to expand the crack opening. The labels 1 mm and 2 mm describe nominal prepared severity states rather than calibrated crack-width measurements; these states were used only to test whether the proposed indicators increase consistently with damage severity.

### 2.4. Signal-Processing Workflow

**Figure 2:**
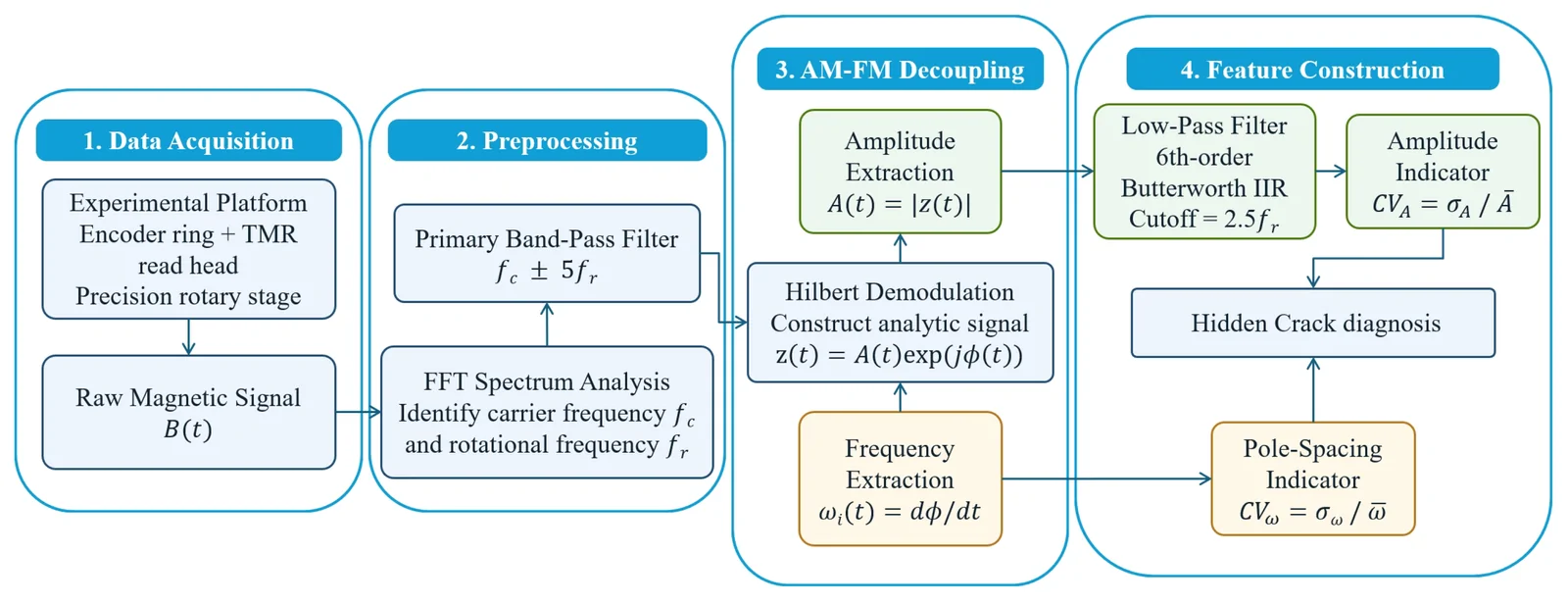
Signal-processing workflow for hidden-crack detection. The raw magnetic signal is processed by FFT-based band-pass filtering, Hilbert transform-based demodulation, extraction of instantaneous amplitude and instantaneous angular frequency, secondary low-pass filtering, and calculation of the normalized indicators ${\mathrm{CV}}_{A}$ and ${\mathrm{CV}}_{\omega }$.

The complete processing pipeline is outlined in Figure 2. It contains four key stages: primary band-pass filtering, Hilbert transform-based demodulation, secondary low-pass filtering, and normalized feature calculation. The workflow combines established ideas from modulation-sideband analysis and envelope-based diagnosis while adapting them to the specific degradation mechanism of magnetic encoder rings [18,19].

In practical operation, the raw magnetic sequence was first inspected in the frequency domain to identify the dominant carrier frequency. A carrier-centered band-pass filter was then applied to remove slow drift and broadband disturbances while retaining the modulation sidebands required for demodulation. The filtered sequence was transformed into an analytic signal using the Hilbert transform, from which the instantaneous amplitude and unwrapped instantaneous phase were obtained. The phase derivative was used to calculate the instantaneous angular frequency, and the demodulated amplitude sequence was further smoothed by the secondary low-pass filter. Finally, the CV was calculated for both sequences so that the resulting indicators described relative fluctuation rather than absolute signal level.

#### 2.4.1. Primary Band-Pass Filtering

The raw magnetic signal contains not only the encoded magnetic waveform but also environmental electromagnetic interference, mechanical vibration, residual eccentricity, and sensor noise. To preserve the fault-sensitive modulation components while removing irrelevant out-of-band disturbances, the signal is first transformed into the frequency domain and processed using an FFT-based band-pass filter. Let $f_{c}$ denote the magnetic carrier frequency identified from the dominant FFT peak and let $f_{r}$ denote the rotational frequency of the encoder ring. Theoretically, for an encoder ring with *N* magnetic poles, the carrier frequency satisfies $f_{c}=Nf_{r}$; for the 32-pole encoder ring used in this study, this gives $f_{c}=32f_{r}$. In the present workflow, a carrier-centered passband is used before Hilbert transform-based demodulation, and the detailed selection and verification of the retained sideband range are discussed in Section 3.1. The angular frequency and ordinary frequency satisfy ω=2πf; therefore, $f_{c}$ and $f_{r}$ are used for frequency-domain filter design, whereas $\omega_{i}\left(t\right)$ denotes the instantaneous angular frequency extracted from the analytic signal.

The primary passband can be generally written as $f_{c}\pm Kf_{r}$, where *K* is the retained rotational sideband order. This normalized form was used because the useful modulation bandwidth scales with rotational speed and pole number through the carrier relation $f_{c}=Nf_{r}$. A small *K* improves noise rejection but may remove crack-sensitive sideband energy, whereas an excessively large *K* admits unrelated high-frequency components and increases demodulation fluctuation. Thus, *K* should be selected from the measured spectrum rather than fixed as an absolute bandwidth in hertz.

#### 2.4.2. Hilbert Transform-Based Demodulation

The Hilbert transform-based demodulation step follows the narrow-band AM–FM assumption stated above: the analyzed signal should be approximated as a single dominant carrier with slowly varying amplitude and phase modulation, and unrelated frequency components should be sufficiently separated from the carrier band [20]. The raw magnetic signal may contain low-frequency drift, residual eccentricity, broadband noise, and environmental interference; therefore, Hilbert transform-based demodulation is applied only after carrier-centered FFT band-pass filtering. In the present experiment, precision rotary control, fixed read-head position, carrier-band selection, and secondary low-pass filtering help satisfy this assumption. For a real signal B(t), its Hilbert transform is defined using the Cauchy principal value (PV) as

(2) $$\hat{B}\left(t\right)=H\;\left\{B(t)\right\}=\frac{1}{\pi }\;\mathrm{PV}\;\int_{-\infty }^{\infty }\frac{B(\tau )}{t-\tau }\;d\tau .$$

where PV denotes the Cauchy principal value. The associated analytic signal is

(3) $$z\left(t\right)=B\left(t\right)+j\hat{B}\left(t\right)=A_{i}\left(t\right)\exp\;\left[j\phi (t)\right].$$

where *j* denotes the imaginary unit and ϕ(t) denotes the instantaneous phase. The instantaneous amplitude and instantaneous angular frequency are then obtained as

(4) $$A_{i}\left(t\right)=\left|z(t)\right|=\sqrt{B^{2}\left(t\right)+{\hat{B}}^{\;2}\left(t\right)}.$$

(5) $$\omega_{i}\left(t\right)=\frac{d\phi (t)}{dt}.$$

In discrete implementation, the wrapped phase was unwrapped to remove ±π discontinuities. Let $\phi_{u}\left[n\right]$ denote the unwrapped phase and Δt=0.1 s denote the sampling interval. The instantaneous angular frequency was calculated by first-order finite difference,

(6) $$\omega_{i}\left[n\right]=\frac{\phi_{u}\left[n+1\right]-\phi_{u}\left[n\right]}{\Delta t}.$$

Because numerical differentiation can amplify high-frequency phase noise, secondary low-pass filtering was used before calculating the CV-based indicators.

#### 2.4.3. Secondary Low-Pass Filtering

Although primary band-pass filtering suppresses most out-of-band disturbances, residual components may remain in the demodulated amplitude sequence. The extracted envelope, namely the instantaneous amplitude $A_{i}\left(t\right)$ defined as the magnitude of the analytic signal in Equation (4), is therefore further processed by a sixth-order Butterworth infinite impulse response (IIR) low-pass filter. After Hilbert transform-based demodulation, the signal of interest is shifted from the carrier region to the low-frequency baseband; thus, the low-pass stage should preserve the modulation band while attenuating residual carrier-related terms and high-frequency artifacts [21].

**Figure 3:**
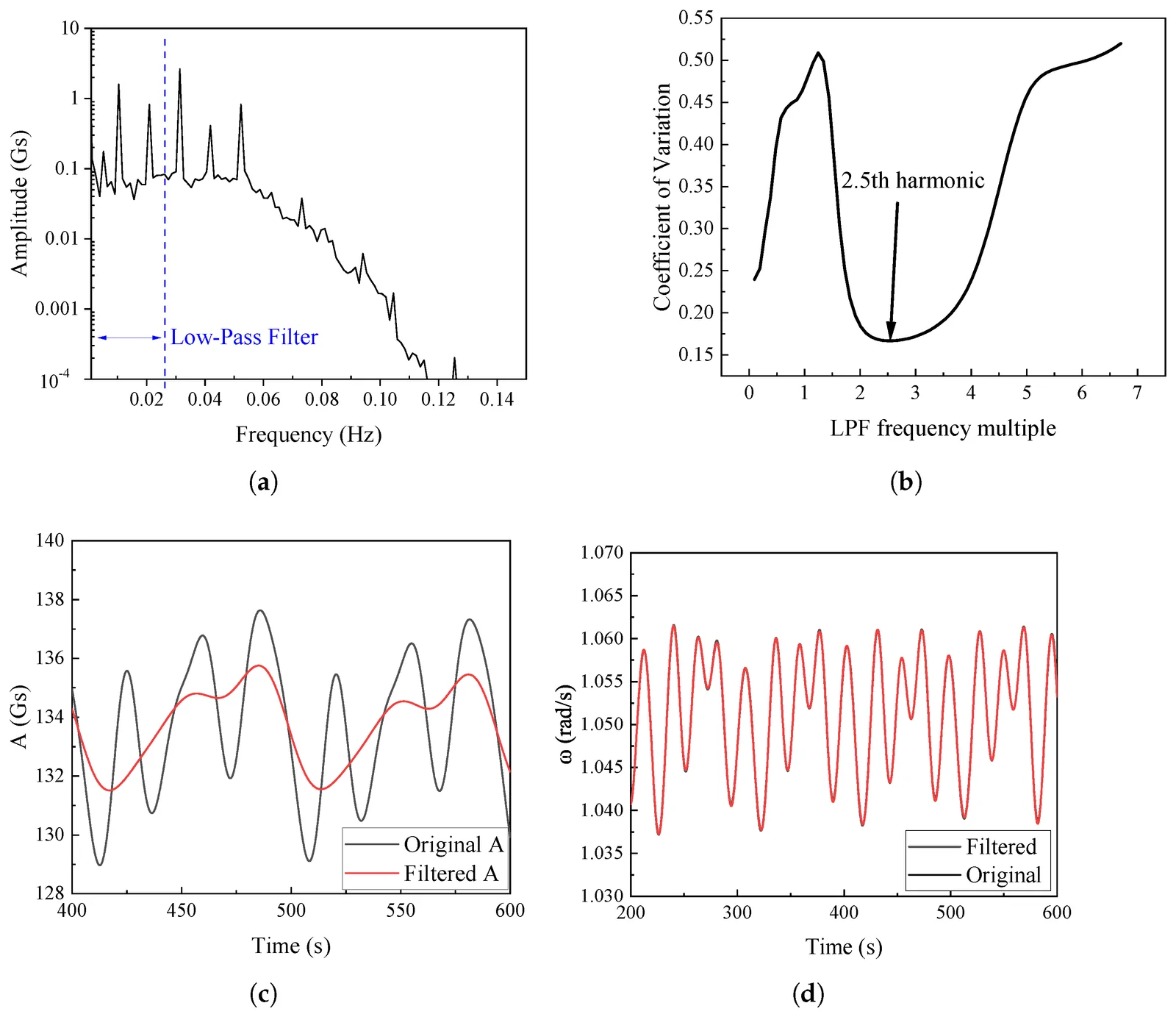
Secondary processing and cutoff-frequency selection for the intact-ring signal. (**a**) Spectrum of the instantaneous-amplitude sequence before secondary low-pass filtering. (**b**) Variation of ${\mathrm{CV}}_{A}$ with candidate low-pass cutoff frequencies, with the minimum obtained at 2.5 $f_{r}$. (**c**) Instantaneous-amplitude sequence before and after sixth-order Butterworth IIR low-pass filtering at 2.5 $f_{r}$. (**d**) Instantaneous-angular-frequency sequence before and after the same low-pass filtering step.

The secondary cutoff was selected by a normalized cutoff-frequency scan, and the cutoff 2.5 $f_{r}$ gave the lowest healthy-state ${\mathrm{CV}}_{A}$ while preserving the main low-frequency modulation trend in the present dataset. The cutoff-frequency selection and the corresponding filtered sequences are shown in Figure 3.

### 2.5. Normalized Feature Construction

The instantaneous-amplitude sequence reflects magnetization uniformity, whereas the instantaneous-angular-frequency sequence reflects pole-spacing consistency. However, the absolute amplitude level is strongly influenced by sensor lift-off distance. To reduce this dependency, two normalized intrinsic indicators are defined using the CV:

(7) $${\mathrm{CV}}_{A}=\frac{\sigma_{A_{i}}}{{\bar{A}}_{i}}.$$

(8) $${\mathrm{CV}}_{\omega }=\frac{\sigma_{\omega_{i}}}{{\bar{\omega }}_{i}}.$$

where $\sigma_{A_{i}}$ and ${\bar{A}}_{i}$ are the standard deviation and mean of the instantaneous-amplitude sequence, and $\sigma_{\omega_{i}}$ and ${\bar{\omega }}_{i}$ are the standard deviation and mean of the instantaneous-angular-frequency sequence. Because the CV is dimensionless, it suppresses the influence of overall signal level and facilitates intrinsic comparison across different sensor lift-off distances. Here, the instantaneous amplitude $A_{i}\left(t\right)$ and instantaneous angular frequency $\omega_{i}\left(t\right)$ are the same quantities defined in Equation (1). The two coefficients, ${\mathrm{CV}}_{A}$ and ${\mathrm{CV}}_{\omega }$, are the final diagnostic indicators used for crack-state identification in this study.

### 2.6. Resolution and Uncertainty Analysis

For the sequential crack-state experiment, the measurement-system magnetic-field resolution was evaluated before calculating the CV indicators. The DAQ6510/7700 acquisition chain (Keithley Instruments/Tektronix, Beaverton, OR, USA) gives a magnetic-field readout resolution of approximately $3.00\times 10^{-3}\;\mathrm{Gs}$, while the calibrated TMR noise gives a noise-limited effective magnetic-field resolution of approximately $4.83\times 10^{-2}\;\mathrm{Gs}$ RMS. Because ${\mathrm{CV}}_{A}$ and ${\mathrm{CV}}_{\omega }$ are nonlinear relative descriptors calculated after filtering, Hilbert demodulation, and normalization by the sequence mean, their uncertainties cannot be represented by a single common instrument-resolution value. The final dataset-specific standard uncertainties were therefore evaluated through the same AM–FM/CV workflow, as reported in Table 1 and detailed in Supplementary Section S3, Tables S3 and S4.

## 3. Results and Analysis

### 3.1. Results for the Intact Encoder Ring

**Figure 4:**
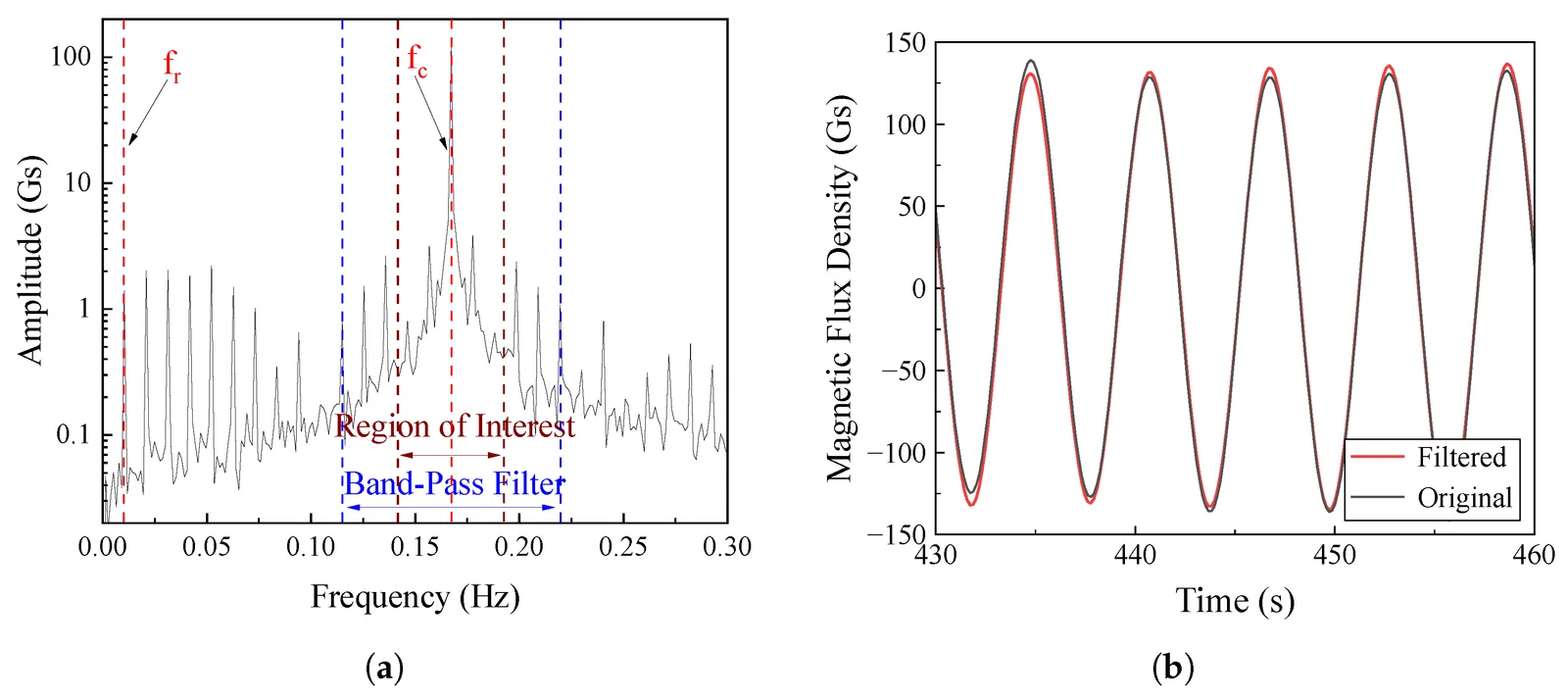
Frequency-domain analysis and primary FFT-based filtering of the intact-ring signal. (**a**) FFT spectrum of the intact-ring raw signal, showing the carrier and low-order rotational sidebands. (**b**) Band-pass-filtered spectrum using the selected passband of $f_{c}\pm 5f_{r}$ to retain the dominant modulation sidebands and suppress outer-band components.

The measured signal of the intact magnetic encoder ring exhibited clear periodicity and was consistent with the compound FM–AM model established in Section 2.1. For the tested 32-pole ring, the theoretical carrier-frequency relation is $f_{c}=32$$f_{r}$, and the corresponding FFT result in Figure 4a shows a dominant carrier peak at the pole-passage frequency surrounded by low-order rotational sidebands. This spectral concentration supports the narrow-band approximation required for Hilbert transform-based demodulation after carrier-centered filtering.

The band-pass-filtered waveform retains the carrier and the most relevant low-order sidebands while suppressing low-frequency eccentricity components and broadband noise. These results confirm that the selected passband preserves the useful modulation content without introducing unnecessary outer-band components. The preprocessing step therefore improves the signal-to-noise ratio and provides a more suitable input for Hilbert transform-based demodulation. The intact ring is used here not only as a healthy reference state but also as a way to verify that preprocessing preserves the nominal encoder waveform rather than amplifying arbitrary noise.

### 3.2. Extraction of Instantaneous Amplitude and Frequency

The derived instantaneous amplitude and instantaneous angular frequency form the basis for intrinsic condition characterization. For the intact encoder ring, the $A_{i}\left(t\right)$ result shown in Figure 3 remains concentrated after filtering, indicating good pole-magnetization uniformity. The companion instantaneous-angular-frequency result $\omega_{i}\left(t\right)$ is obtained from the phase derivative in Equation (5) using the same preprocessed intact signal; it remains close to the nominal pole-passage angular frequency and shows only weak fluctuation, indicating regular pole spacing. This combined behavior of stable $A_{i}\left(t\right)$ and stable $\omega_{i}\left(t\right)$ is expected for a healthy encoder ring without observable local damage. It also provides the healthy-state baseline required by the AM–FM model: when the pole array has no intentional local damage, both the amplitude-related and frequency-related quantities remain comparatively stable after demodulation.

The two extracted sequences provide complementary information. The instantaneous-amplitude sequence is directly affected by the local magnetic-field strength sensed by the read head and is therefore sensitive to magnetization attenuation or field redistribution near a damaged pole. The instantaneous-angular-frequency sequence is obtained from the phase evolution and is more closely related to the regularity of pole passage. A hidden crack may affect both quantities, but the relative change can differ depending on whether the dominant consequence is local weakening of magnetization, local pole-shape distortion, or a combined change in the field distribution. Using both sequences therefore reduces the risk of missing a weak defect that is visible mainly in only one modulation component.

### 3.3. Effect of Sensor Lift-Off Distance

**Figure 5:**
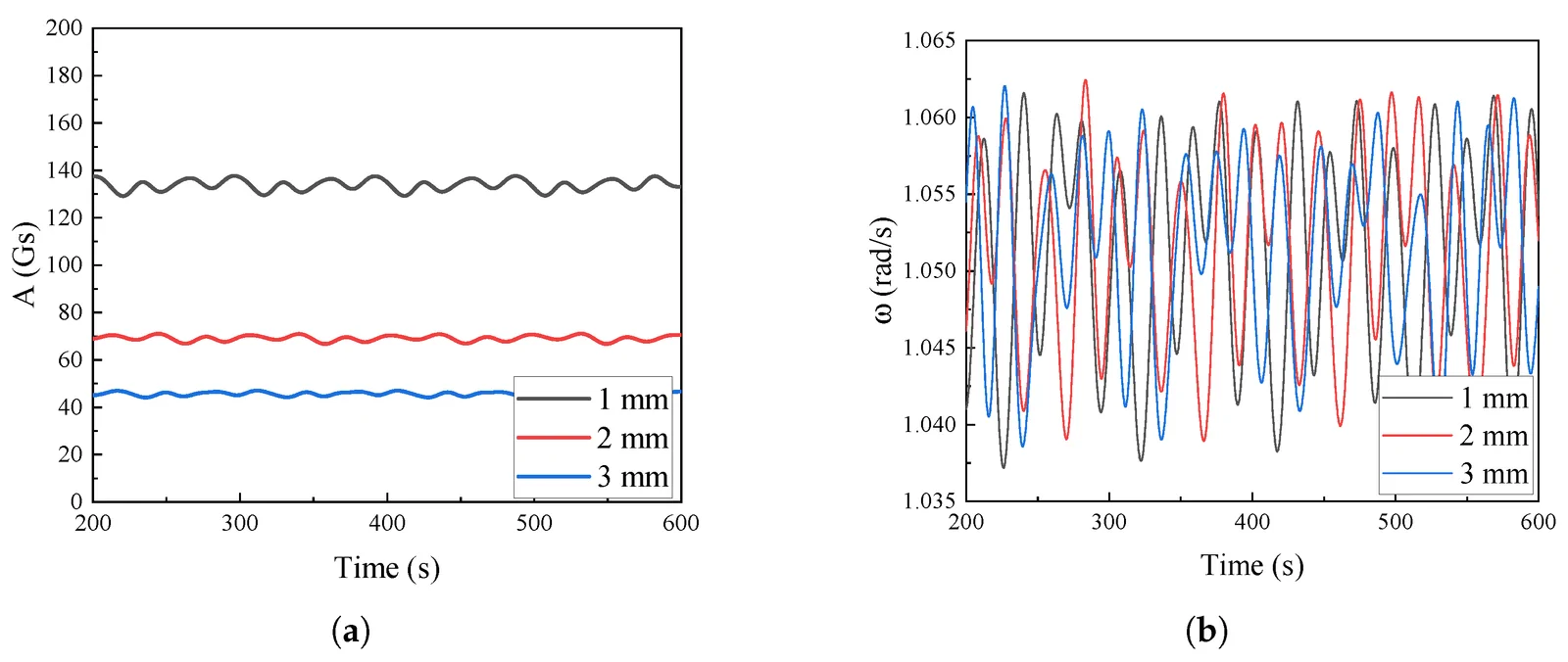
Influence of sensor lift-off distance on the extracted feature sequences. (**a**) Filtered instantaneous-amplitude sequences measured at lift-off distances of 1, 2, and 3 mm. (**b**) Instantaneous-angular-frequency sequences measured under the same lift-off-distance conditions.

Figure 5 compares the processed results obtained at sensor lift-off distances of 1, 2, and 3 mm. As the sensor lift-off distance increases, the absolute signal amplitude decreases because of spatial magnetic-field attenuation. Consequently, both the mean level and the standard deviation of the amplitude-related sequence exhibit clear distance dependence. In contrast, the instantaneous angular-frequency sequence remains much less sensitive to sensor lift-off distance.

After normalization by the CV, the distance dependence is markedly reduced. Across the three tested sensor lift-off distances, the amplitude-related CV values remain within a narrow fluctuation band, with dispersions on the order of 0.016–0.017, whereas the frequency-related CV remains close to 0.006. These results indicate that ${\mathrm{CV}}_{A}$ and ${\mathrm{CV}}_{\omega }$ can serve as intrinsic indicators that are substantially less sensitive to sensor lift-off distance after normalization and are therefore more suitable for diagnosis than unnormalized statistics. In this respect, the proposed indicators complement prior encoder-signal health measures by providing a more robust representation of magnetic-pole degradation.

The same processing workflow was also applied to supplementary records from three other magnetic encoder rings under three measured speed conditions. The raw flux-density traces, extracted CV indicators, and complete intermediate processing records are provided in Supplementary Section S1, Table S1 and Figures S1–S10 rather than added as extra main-text figures or tables.

## 4. Detection of Hidden Cracks

To verify sensitivity to early hidden-crack damage, a single-variable comparative experiment was designed using the same encoder ring under four states: intact, hidden crack, visible 1 mm crack, and visible 2 mm crack. The preparation procedure for these states is described in Section 2.3.

Using the same ring and measurement platform for the four states reduces the influence of specimen-to-specimen variation and highlights the effect of progressive damage. The intact state provides the baseline fluctuation level of the magnetic pole array, whereas the hidden-crack state represents the target early fault condition. The two visible-crack states are not the primary detection target, but they provide a severity reference for evaluating whether the proposed indicators respond consistently as damage becomes more pronounced. This arrangement makes it possible to examine both early detectability and trend monotonicity within the same experimental framework.

**Figure 6:**
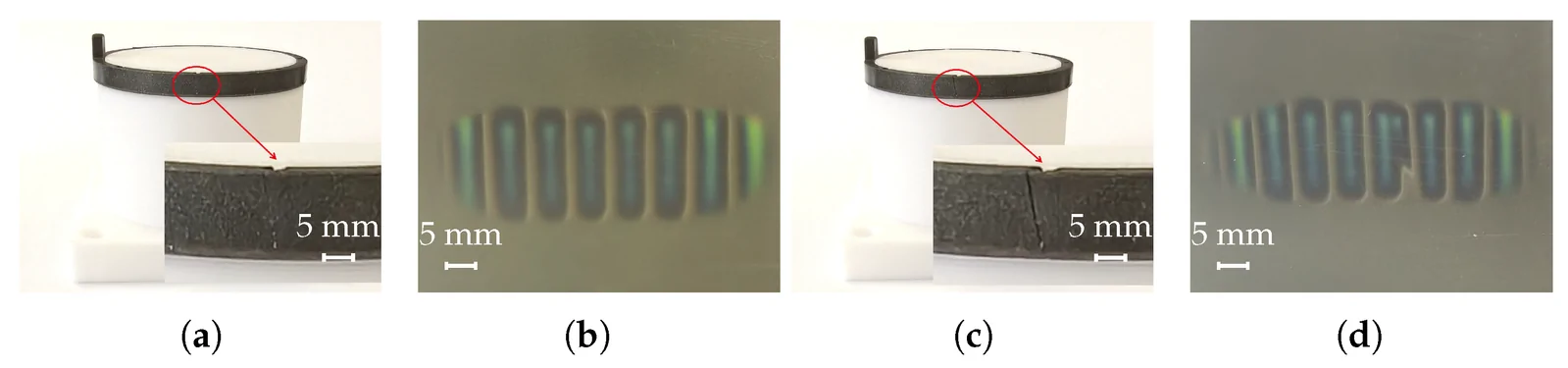
Visual and magnetic-viewing observations of crack states. Approximate scale bars indicate 5 mm. (**a**) Photograph of the prepared hidden-crack region. (**b**) Magnetic-viewing-film observation of the same hidden-crack region. (**c**) Photograph of the visible-crack state. (**d**) Magnetic-viewing-film observation of the visible-crack state.

Representative photographs and magnetic viewing observations of the crack specimens are shown in Figure 6. For the hidden-crack specimen, the damage remains difficult to identify by direct visual inspection or by magnetic viewing, whereas the visible-crack specimen provides a comparative reference with a clearly observable defect. This comparison motivates the subsequent signal-based diagnosis route.

**Figure 7:**
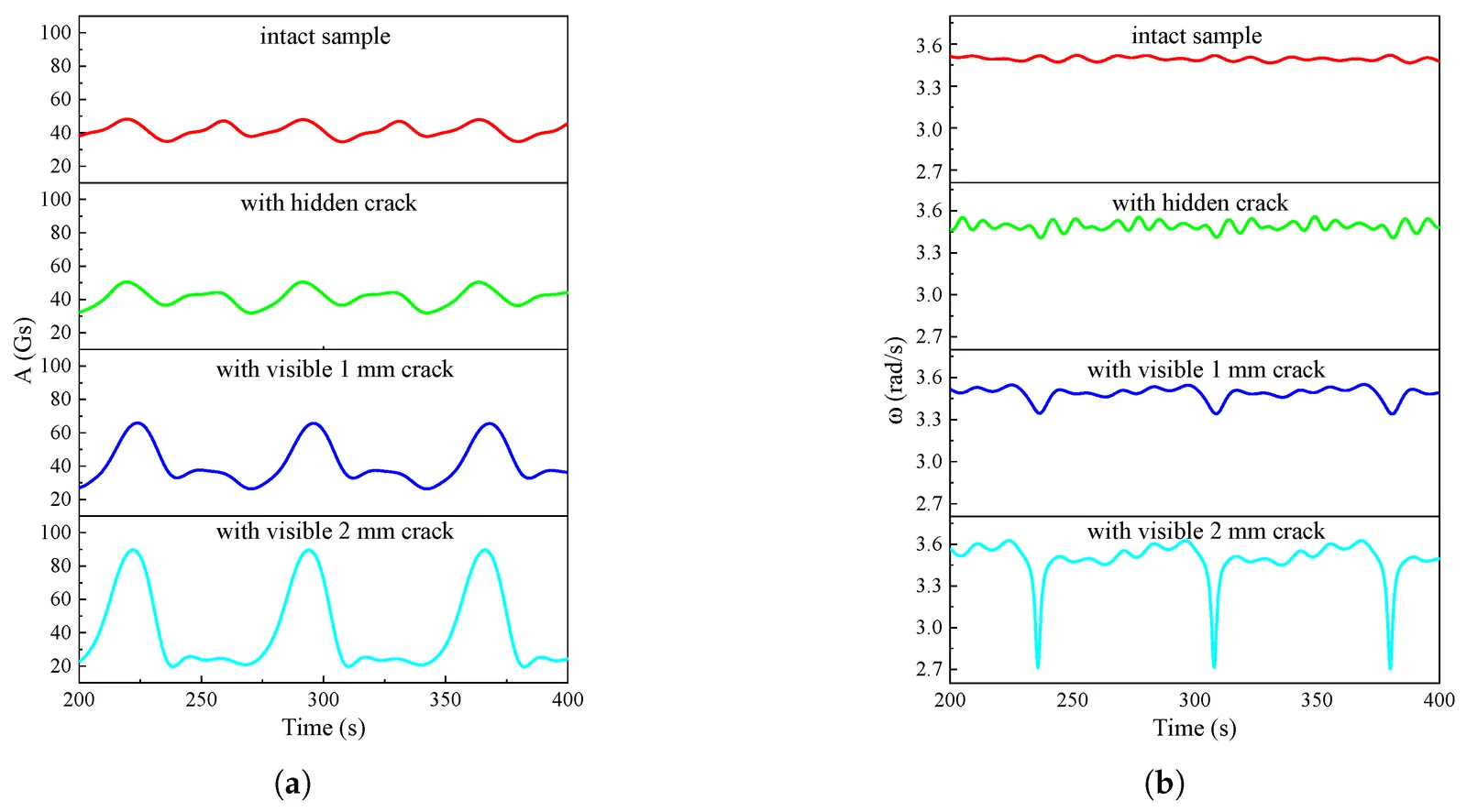
Demodulated feature responses under different damage states. (**a**) Instantaneous-amplitude sequences for the intact ring, hidden crack, visible 1 mm crack, and visible 2 mm crack. (**b**) Instantaneous-angular-frequency sequences for the same four states.

Figure 7 compares the instantaneous-amplitude and instantaneous-angular-frequency responses for the four damage states. Figure 7a summarizes the instantaneous-amplitude sequences for the intact ring, hidden crack, visible 1 mm crack, and visible 2 mm crack, whereas Figure 7b summarizes the corresponding instantaneous-angular-frequency sequences. The intact ring exhibits a stable amplitude envelope and an almost constant instantaneous angular frequency. Once a hidden crack appears, weak but repeatable amplitude fluctuation and frequency deviation become observable. As the damage develops into visible cracks of 1 mm and 2 mm, both the waveform distortion and the fluctuation intensity increase markedly.

These experimental observations provide direct support for the AM–FM physical interpretation. The transition from the intact state to the hidden-crack state produces simultaneous but weak changes in both the instantaneous-amplitude and instantaneous-angular-frequency sequences, indicating that even an incipient crack affects both local field strength and pole-passage regularity. As the crack opening is enlarged to the visible 1 mm and 2 mm states, the stronger amplitude fluctuation and the accompanying increase in frequency fluctuation agree with the model prediction that progressive pole degradation is expressed as coupled AM and FM perturbations.

The quantitative results are summarized in Table 1. These values should be interpreted as relative indicators obtained under controlled and comparable conditions, rather than as universal alarm thresholds. The intact encoder ring exhibits very low values of both indicators, with ${\mathrm{CV}}_{A}=0.092$ and ${\mathrm{CV}}_{\omega }=0.004$. In the hidden-crack state, the indicators increase to 0.127 and 0.008, respectively. For the nominal visible 1 mm and 2 mm states, both indicators increase further, reaching ${\mathrm{CV}}_{A}=0.290$ and 0.593, and ${\mathrm{CV}}_{\omega }=0.013$ and 0.039.

These results indicate that the proposed indicators are sensitive to incipient degradation and maintain a generally positive relationship with damage severity. They are therefore suitable not only for early warning but also for trend monitoring of hidden-crack evolution.

From a diagnostic perspective, the most important comparison is between the intact and hidden-crack states. The increase in ${\mathrm{CV}}_{A}$ from 0.092 to 0.127 and the increase in ${\mathrm{CV}}_{\omega }$ from 0.004 to 0.008 show that the hidden crack produces measurable modulation irregularity even when direct observation remains unreliable. The larger increases observed for the visible 1 mm and 2 mm cracks further indicate that the indicators are not merely binary fault labels; they also carry information about the degree of degradation. This behavior is favorable for practical monitoring, where an early warning should ideally be followed by continued tracking of damage development.

## 5. Discussion

This study modeled the magnetic signal of a magnetic encoder ring as a compound FM–AM waveform and used Hilbert transform-based demodulation with CV normalization to diagnose early hidden cracks. The results show that the two extracted indicators describe different but complementary aspects of pole-array degradation: ${\mathrm{CV}}_{A}$ reflects relative instantaneous-amplitude nonuniformity associated with local magnetization changes, whereas ${\mathrm{CV}}_{\omega }$ reflects phase-derived pole-passage irregularity. The discussion below focuses on indicator selection, possible error sources, practical use, future work, and the advantages of the proposed framework.

### 5.1. Why CV Was Selected as the Indicator

The CV was selected because the diagnostic objective is to quantify relative nonuniformity rather than absolute signal magnitude. By normalizing fluctuation by the mean of the same sequence, ${\mathrm{CV}}_{A}$ describes instantaneous-amplitude nonuniformity and ${\mathrm{CV}}_{\omega }$ describes phase-derived pole-passage regularity under controlled but not perfectly identical signal amplitudes. The lift-off experiment in Figure 5 supports this choice because the normalized indicators vary much less than the absolute amplitude level. A descriptor-level comparison supporting this selection is provided in Supplementary Section S2 and Table S2.

### 5.2. Practical Application Issues

In the present implementation, the retained passband was selected from the measured spectral distribution rather than assigned only as an empirical parameter. FFT inspection showed that hidden-crack-related modulation was mainly located in the low-order rotational sidebands around the carrier, and the spectral peak at the fifth rotational sideband was approximately 2% of the carrier amplitude. Therefore, the FFT-based band-pass filter was extended to $f_{c}\pm 5$$f_{r}$ to retain crack-sensitive low-order sidebands while excluding weak outer-band components. The proposed method provides a feasible route for practical monitoring, but alarm thresholds should not be treated as universal constants. For deployment, a healthy baseline should first be collected for each magnetic encoder ring configuration, and warning limits should be calibrated from baseline dispersion or from measured separation between healthy and defective states. Additional requirements such as robustness under strong temperature drift, structural vibration, and electromagnetic interference depend on specific field conditions and therefore require application-specific validation beyond the present controlled laboratory study. The supplementary multi-speed tests show that the CV of $A_{i}$ and the CV of $\omega_{i}$ remain similar under moderate speed changes for three other magnetic encoder rings, as summarized in Supplementary Section S1 and Table S1. Therefore, when a known periodic vibration or electromagnetic disturbance overlaps a diagnostic band, the rotational speed can be adjusted to shift the pole-passage carrier and crack-sensitive sidebands away from the disturbance frequency, making subsequent band-pass or low-pass filtering more effective. The disturbance-separation example in Supplementary Section S4, Figure S11, and Table S5 illustrates this strategy, although direct field disturbance tests are still needed before industrial deployment.

For an analysis window containing *N* samples, the dominant computational steps are the FFT-based spectral/passband operations and the FFT-based Hilbert transform, each with O(NlogN) complexity. The Butterworth IIR low-pass filtering, phase unwrapping and finite-difference calculation, and CV calculation are linear operations with O(N) complexity. Therefore, the overall workflow has O(NlogN) time complexity and O(N) memory usage. In the present crack-state records, the 10 Hz sampling rate and 0.625 rpm rotation give approximately 960 samples per revolution and 6720 samples for the seven-revolution analysis window, which is a small workload for an embedded processor or industrial PC. For online monitoring, the algorithm can be updated after each complete-revolution window or by using a sliding complete-revolution window. The practical latency is therefore governed mainly by the selected window length rather than by computation time: a shorter one- or several-revolution window enables faster alarms, whereas a longer window improves repeatability.

### 5.3. Possible Error Sources and Influencing Factors

The main influencing factors are residual eccentricity, rotary-stage stability, sensor lift-off distance, phase noise, environmental disturbances, and the nominal preparation of visible crack states. Residual eccentricity and lift-off variation mainly affect the amplitude-related sequence and are expected to have a moderate influence when the installation changes between measurements; in the present fixed setup their effect is reduced by alignment and CV normalization. Rotary-stage instability and phase noise mainly affect $\omega_{i}\left(t\right)$ and ${\mathrm{CV}}_{\omega }$; their influence is expected to be small under the precision rotary stage used here but may increase under unstable speed control. Electromagnetic interference and structural vibration can have a large influence if their frequencies overlap the retained carrier band or low-frequency modulation band, which is why speed selection and filtering should be configured for each application. The visible-crack labels of 1 mm and 2 mm should be understood as qualitative severity labels produced by filler-induced opening, not as calibrated crack-width measurements. The measurement-system magnetic-field resolution is common to the four crack-state records, but the uncertainty of each CV result depends on the processed sequence, the local signal level, the fluctuation amplitude, filtering response, and sample count. Therefore, the final dataset-specific CV standard uncertainties are included in Table 1; the equipment inputs, resolution calculation, and window-level CV uncertainty propagation are provided in Supplementary Section S3, Tables S3 and S4.

### 5.4. Future Research Directions

Future work should expand the database of fault modes and specimens, especially to distinguish hidden cracks from partial demagnetization, debris contamination, and other early magnetic faults. The filtering parameters should also be made adaptive to pole number, speed, sensor bandwidth, and noise environment. Finally, the proposed interpretable indicators can be combined with learning-based classifiers or inverse error models so that early degradation monitoring can be integrated with online compensation and health-aware motion control.

### 5.5. Advantages of the Proposed Framework

The main advantage of the proposed framework is its physical interpretability. Unlike compensation-oriented approaches that mainly correct encoder output after error formation [22,23], the present method links signal modulation directly to magnetic-pole degradation. It also avoids reliance on a high-accuracy external reference encoder, which distinguishes it from calibration-heavy angle-error evaluation procedures [24,25]. Compared with general data-driven structural health monitoring models, which often require training datasets and iterative model inference, the present method is simpler to implement for controlled magnetic encoder ring monitoring because the dominant operations are FFT-based filtering, Hilbert transform-based demodulation, low-pass filtering, and CV calculation. These properties make the framework useful as an early-warning layer, while broader statistical validation remains necessary before universal thresholds can be recommended.

## 6. Conclusions

This study investigated early hidden-crack diagnosis in magnetic encoder rings by modeling the acquired magnetic signal as a compound FM–AM waveform and decoupling its amplitude- and frequency-related components. A precision-controlled TMR-based measurement platform and a signal-processing workflow combining carrier-centered band-pass filtering, Hilbert transform-based demodulation, secondary low-pass filtering, and CV analysis were developed. Under fixed and comparable measurement conditions, ${\mathrm{CV}}_{A}$ and ${\mathrm{CV}}_{\omega }$ quantified magnetization uniformity and pole-passage regularity, respectively. Comparative experiments on intact, hidden-crack, and nominal visible-crack states demonstrated that the indicators can detect weak hidden-crack-related modulation irregularity and increase consistently with prepared damage severity. The method is interpretable, does not require a high-accuracy external reference encoder, and has computational characteristics suitable for online monitoring; however, practical thresholds and robustness under stronger environmental disturbances should be calibrated and validated for each application scenario.

## Figures and Tables

**Table 1:** Quantitative comparison of the proposed CV-based indicators under different damage states. The listed values are dimensionless CV indicators calculated with the same AM–FM processing workflow and reported as value ± standard uncertainty; each uncertainty is rounded to one significant digit.

Damage State	${\mathrm{CV}}_{A}$	${\mathrm{CV}}_{\omega }$
Intact	0.092±0.001	0.0044±0.0001
Hidden crack	0.127±0.005	0.0080±0.0002
Visible 1 mm crack	0.290±0.004	0.013±0.001
Visible 2 mm crack	0.593±0.004	0.04±0.01

## Data Availability

The original contributions presented in this study are included in the article/Supplementary Materials. Further inquiries can be directed to the corresponding author.

## Supplementary Materials

The following supporting information can be downloaded at: https://www.mdpi.com/article/10.3390/s1010000/s1, Figure S1: Raw magnetic flux-density signals for magnetic rings A, B, and C at three measured rotational speeds; Figures S2–S10: Intermediate AM–FM/CV processing results for rings A, B, and C at the three measured speeds; Figure S11: Disturbance-separation validation by speed adjustment; Table S1: Supplementary validation across magnetic rings and measured fundamental rotational frequencies; Table S2: Comparison of candidate descriptors for the diagnostic sequences; Table S3: Equipment inputs used in the magnetic-field resolution calculation; Table S4: Window-level CV values and uncertainty calculation for the four crack states; Table S5: CV indicators for the disturbance-separation validation.
