# Supplementary material for: Early Hidden-Crack Detection in Circular Magnetic Encoder Rings Through FM-AM Signal Decoupling
Source: Sensors (Basel). 2026 Aug 14;26(16):5135. doi: 10.3390/s26165135 (PMC13517416; doi:10.3390/s26165135)
Supplement: Supplementary file 1 [file sensors-26-05135-s001.zip › sensors-4408467-supplementary.pdf]

Supplementary Materials for
Early Hidden-Crack Detection in Circular Magnetic Encoder Rings through FM-AM Signal Decoupling

S1. Additional Multi-Ring and Speed-Variation Validation

This section supports the robustness discussion in Sections 3.3 and 5.2 by testing whether the same AM–FM/CV workflow remains applicable to different encoder rings operated at different rotational speeds. The specific purpose is to verify that the two proposed indicators, CV_A and CV_ω, remain mainly ring-dependent rather than speed-dependent when the same ring is measured at several speeds. Additional records were acquired from magnetic rings A, B, and C under three measured fundamental rotational frequencies. The command strings in the raw file names were used only as acquisition identifiers; speed was reported from the measured rotational fundamental frequency f_r. The numerical values in the raw files were converted from voltage to magnetic flux density using 3.3 mV/Gs before processing.

Table S1: Supplementary validation across magnetic rings and measured fundamental rotational frequencies. The uncertainty of each CV value is the standard uncertainty estimated from seven complete-revolution windows and rounded to one significant digit. The rotational speed was calculated as $60 f_r$ rpm.

Ring	f_r (Hz)	Speed (rpm)	f_c (Hz)	f_c/f_r	K	$\mathrm{CV}_A \pm u$	$\mathrm{CV}_\omega \pm u$
A	0.042	2.51	0.670	16.00	5.00	0.17 ± 0.02	0.013 ± 0.005
A	0.069	4.17	1.111	16.00	5.00	0.17 ± 0.04	0.007 ± 0.004
A	0.098	5.87	1.566	16.00	5.00	0.17 ± 0.01	0.007 ± 0.004
B	0.042	2.51	0.419	10.00	4.50	0.15 ± 0.01	0.016 ± 0.005
B	0.069	4.17	0.695	10.00	4.50	0.15 ± 0.02	0.016 ± 0.007
B	0.098	5.87	0.978	10.00	4.50	0.15 ± 0.01	0.013 ± 0.003
C	0.042	2.51	0.168	4.00	1.80	0.024 ± 0.003	0.013 ± 0.003
C	0.069	4.17	0.278	4.00	1.80	0.026 ± 0.005	0.014 ± 0.006
C	0.098	5.87	0.391	4.00	1.80	0.023 ± 0.002	0.011 ± 0.003

Each record was processed using the same AM–FM workflow as the main text. First, the raw sequence was interpolated onto a uniform time grid and truncated to complete revolutions. Second, the dominant carrier frequency f_c was identified from the raw spectrum. Third, an FFT-based primary band-pass filter was applied around the carrier as $f_c \pm K f_r$. Because the supplementary magnetic rings have lower carrier orders than the main 32-pole ring, K was selected from the measured spectrum while preventing the lower passband edge from entering the baseband; this preserves the carrier-neighborhood sidebands required for Hilbert demodulation. Fourth, the analytic signal was calculated by the Hilbert transform, and the instantaneous amplitude and instantaneous angular frequency were extracted. Finally, a secondary low-pass filter with cutoff $2.5 f_r$ was applied

before calculating CV_A and CV_ω. The equivalent angular error was estimated from the carrier-phase residual after removing the best-fit linear phase trend and dividing the residual phase by the carrier order f_c/f_r.

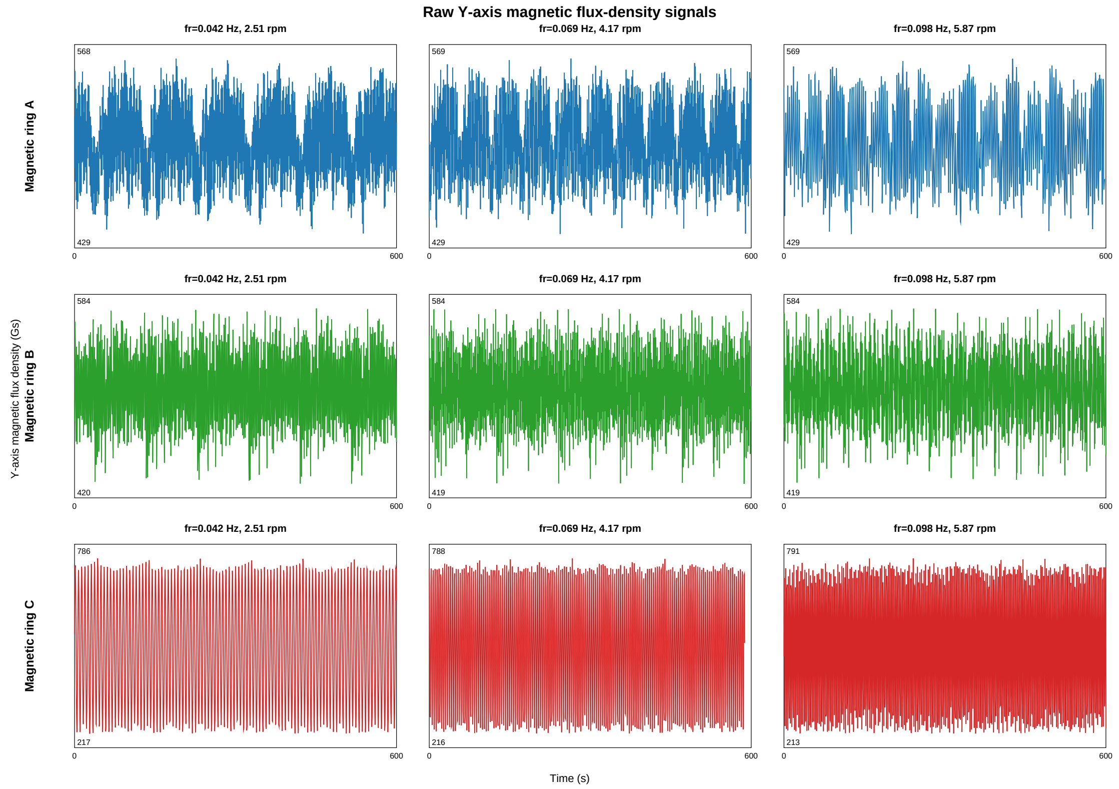

Figure S1: Raw Y-axis magnetic flux-density signals of magnetic rings A, B, and C under three measured fundamental rotational frequencies.

The purpose of this comparison is to show that the proposed method and processing workflow remain usable for different magnetic rings and rotational speeds. The evidence is that, for each magnetic ring, the two proposed CV indicators remain approximately stable when only the speed is changed, whereas the carrier frequency f_c shifts proportionally with f_r. For CV_A, the maximum relative spread across the three speeds is approximately 2.5% for magnetic ring A, 1.9% for magnetic ring B, and 11.1% for magnetic ring C. For CV_ω, the absolute ranges are also small: 0.0055 for ring A, 0.0038 for ring B, and 0.0035 for ring C. These results indicate that the indicators are primarily associated with the magnetic ring condition and are not simply artifacts of the selected rotational speed. Thus, changing the speed shifts the spectral position of the carrier but does not invalidate the AM–FM/CV workflow. Therefore, when a known periodic disturbance is present in practical operation, the rotational speed can be selected so that the pole-passage carrier and its crack-sensitive sidebands are separated from the disturbance frequency before the same filtering and demodulation workflow is applied.

Figures S2–S10 show the complete processing record for each dataset. In each figure, the six panels correspond to the raw magnetic flux-density signal, the raw spectrum and selected primary passband, the primary band-pass output, the instantaneous amplitude before and after secondary low-pass filtering, the instantaneous angular frequency before and after secondary low-pass filtering, and the equivalent angular-error sequence.

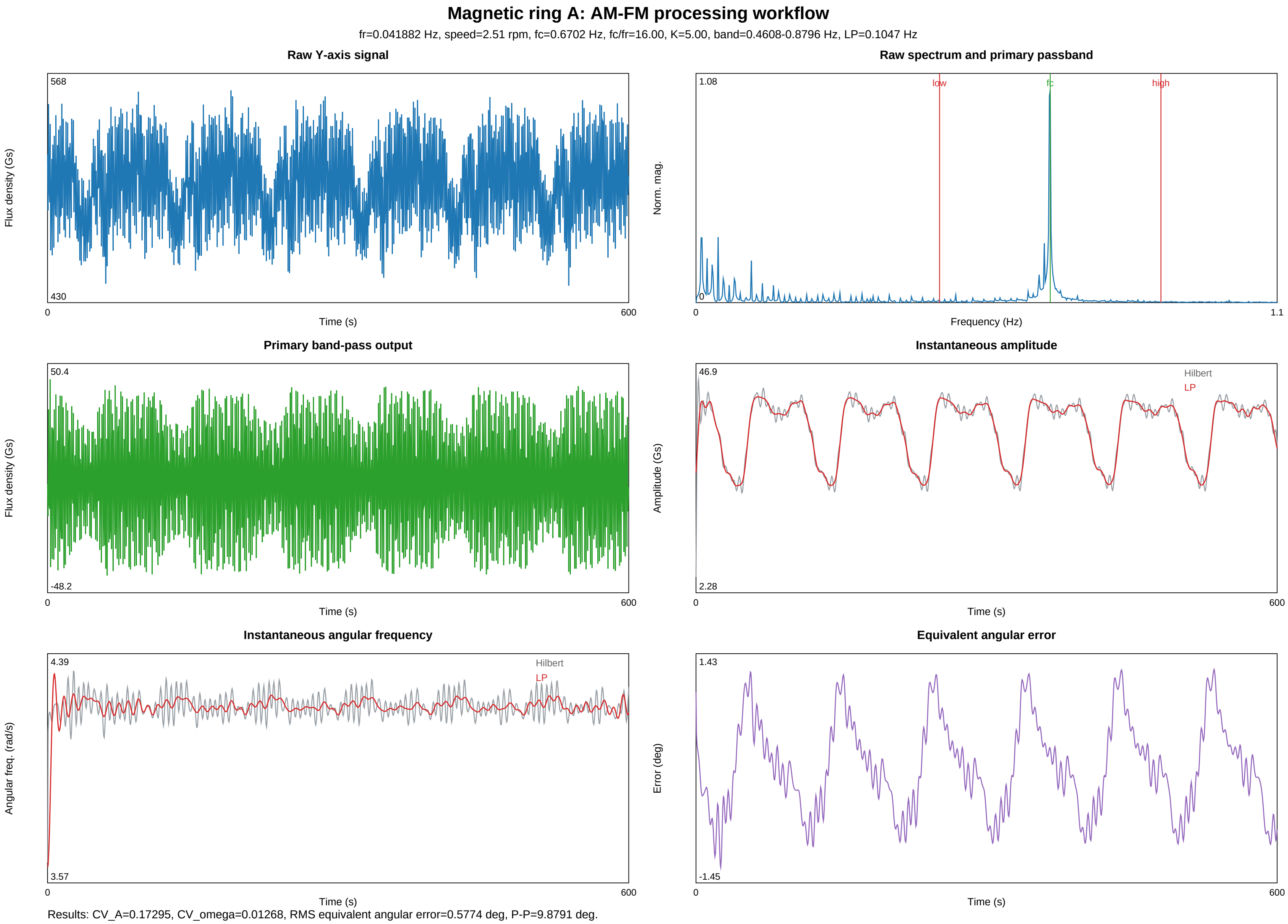

Figure S2: Processing details for magnetic ring A at $f_r = 0.042$ Hz.

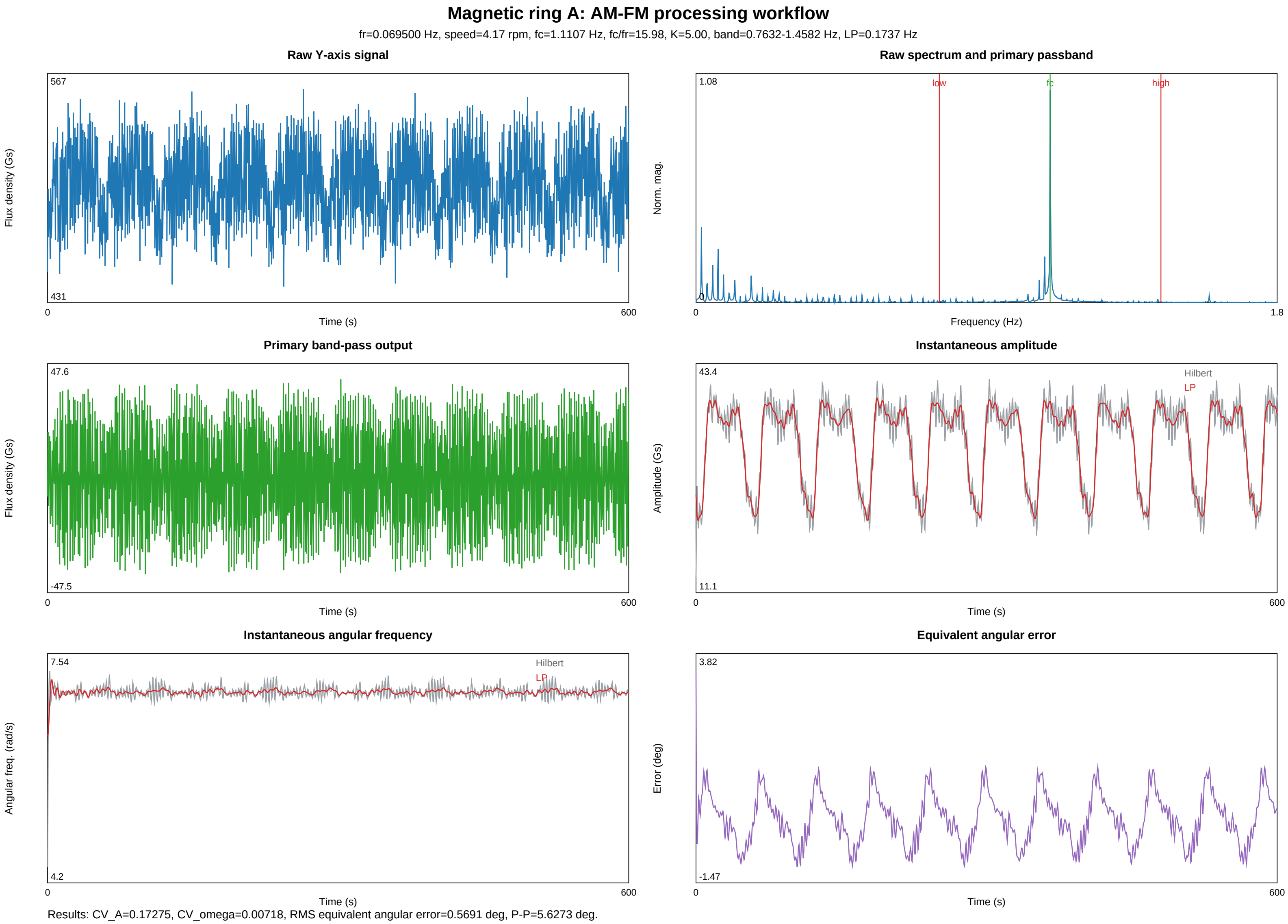

Figure S3: Processing details for magnetic ring A at $f_r = 0.069$ Hz.

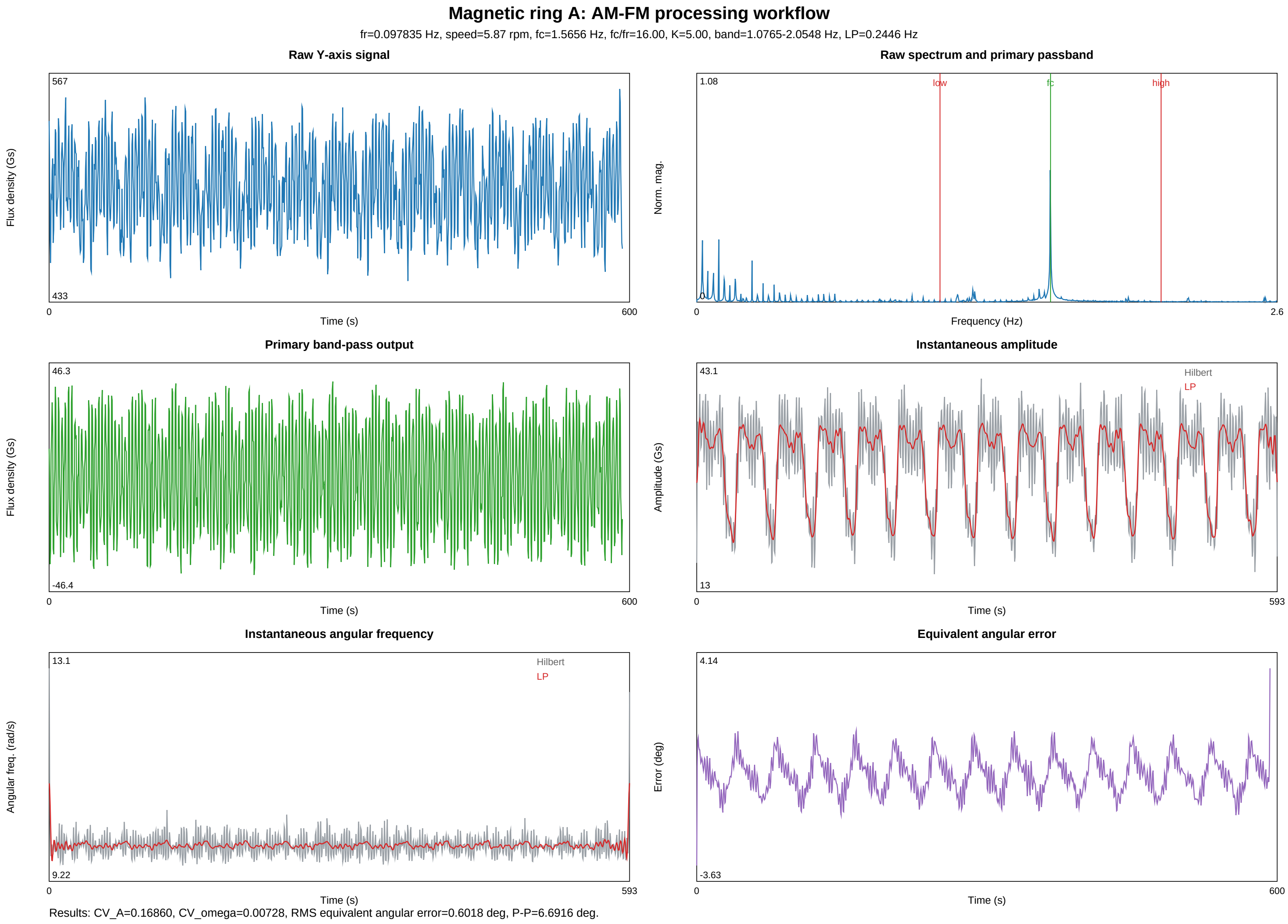

Figure S4: Processing details for magnetic ring A at $f_r = 0.098$ Hz.

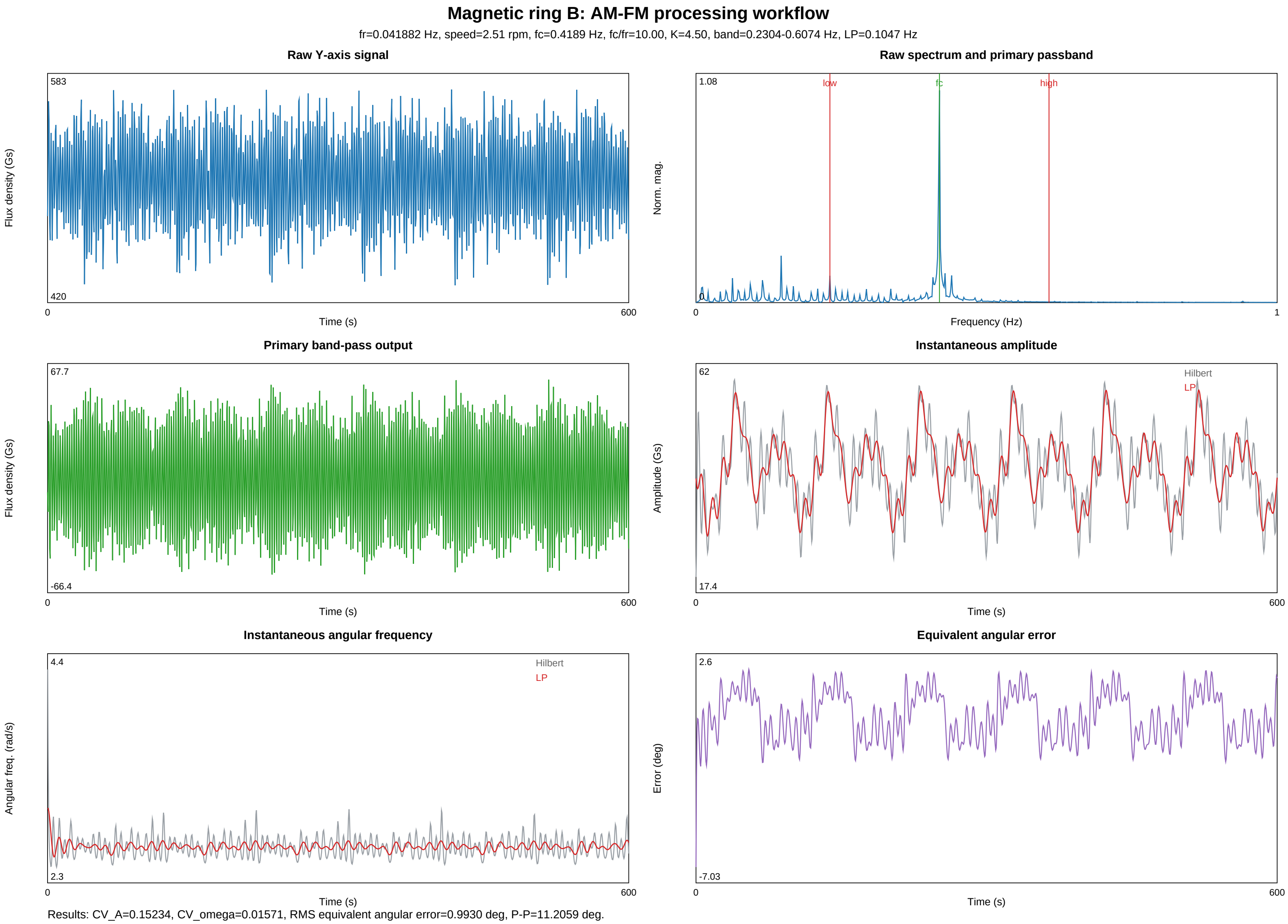

Figure S5: Processing details for magnetic ring B at $f_r = 0.042$ Hz.

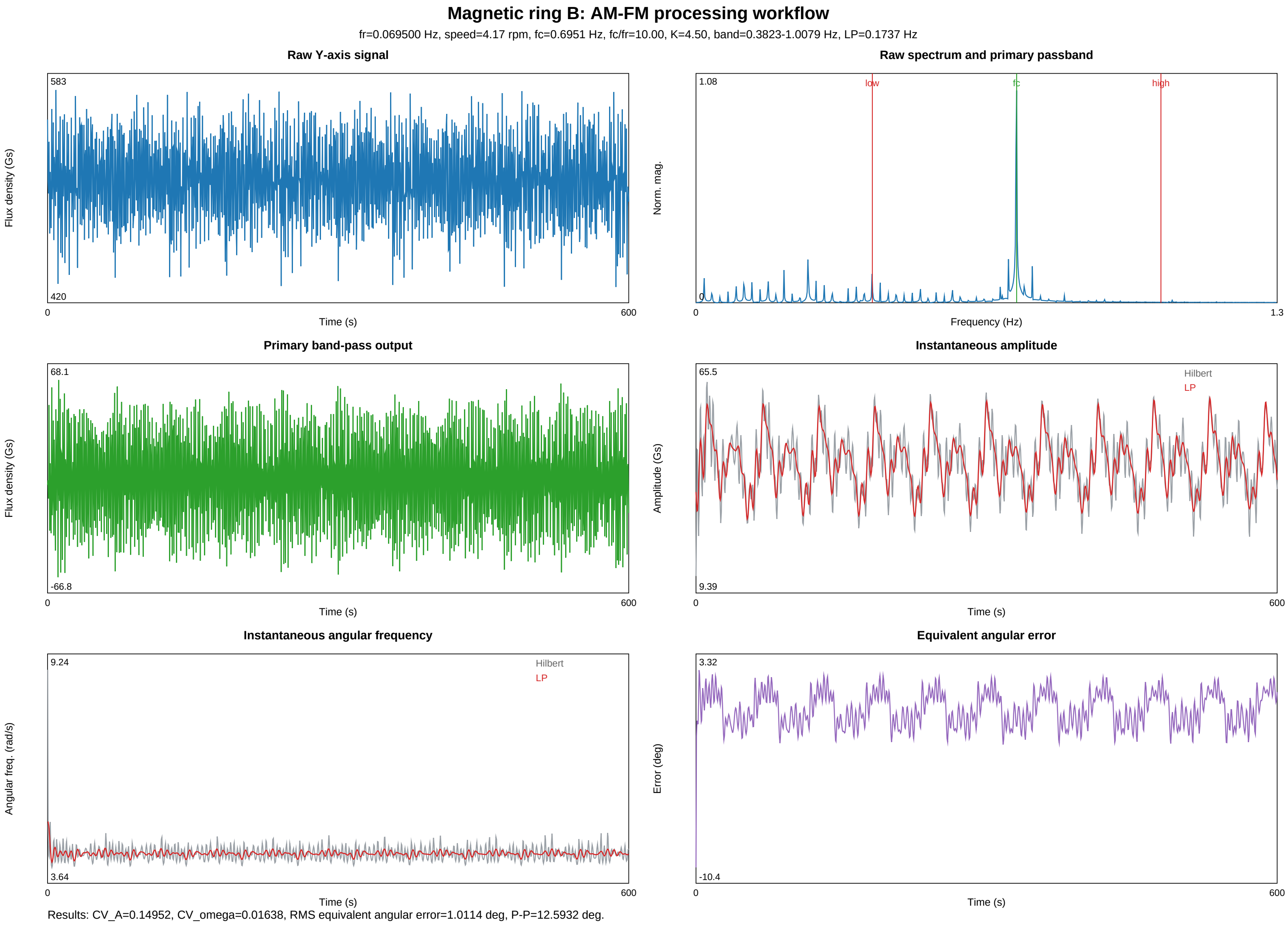

Figure S6: Processing details for magnetic ring B at $f_r = 0.069$ Hz.

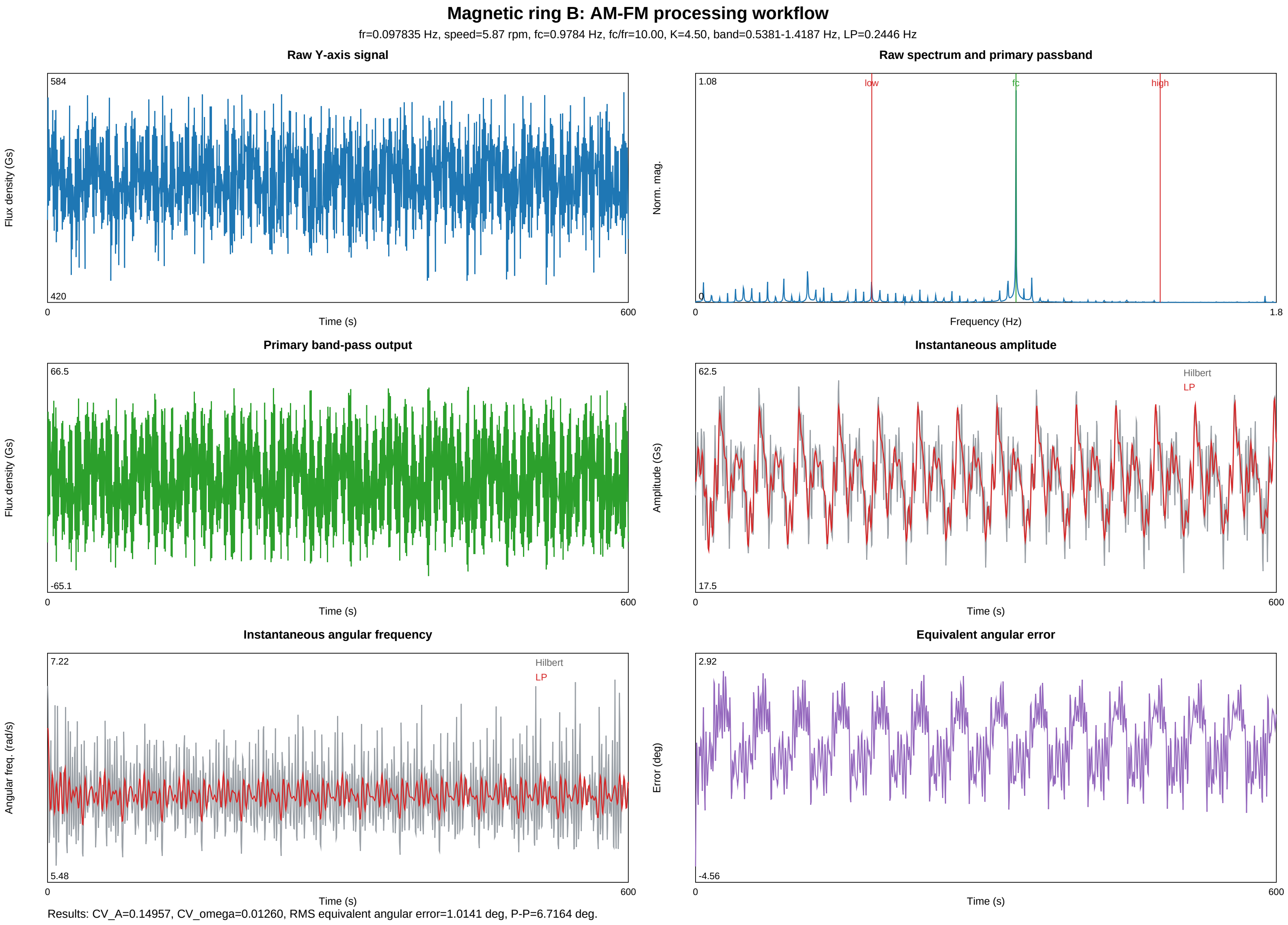

Figure S7: Processing details for magnetic ring B at $f_r = 0.098$ Hz.

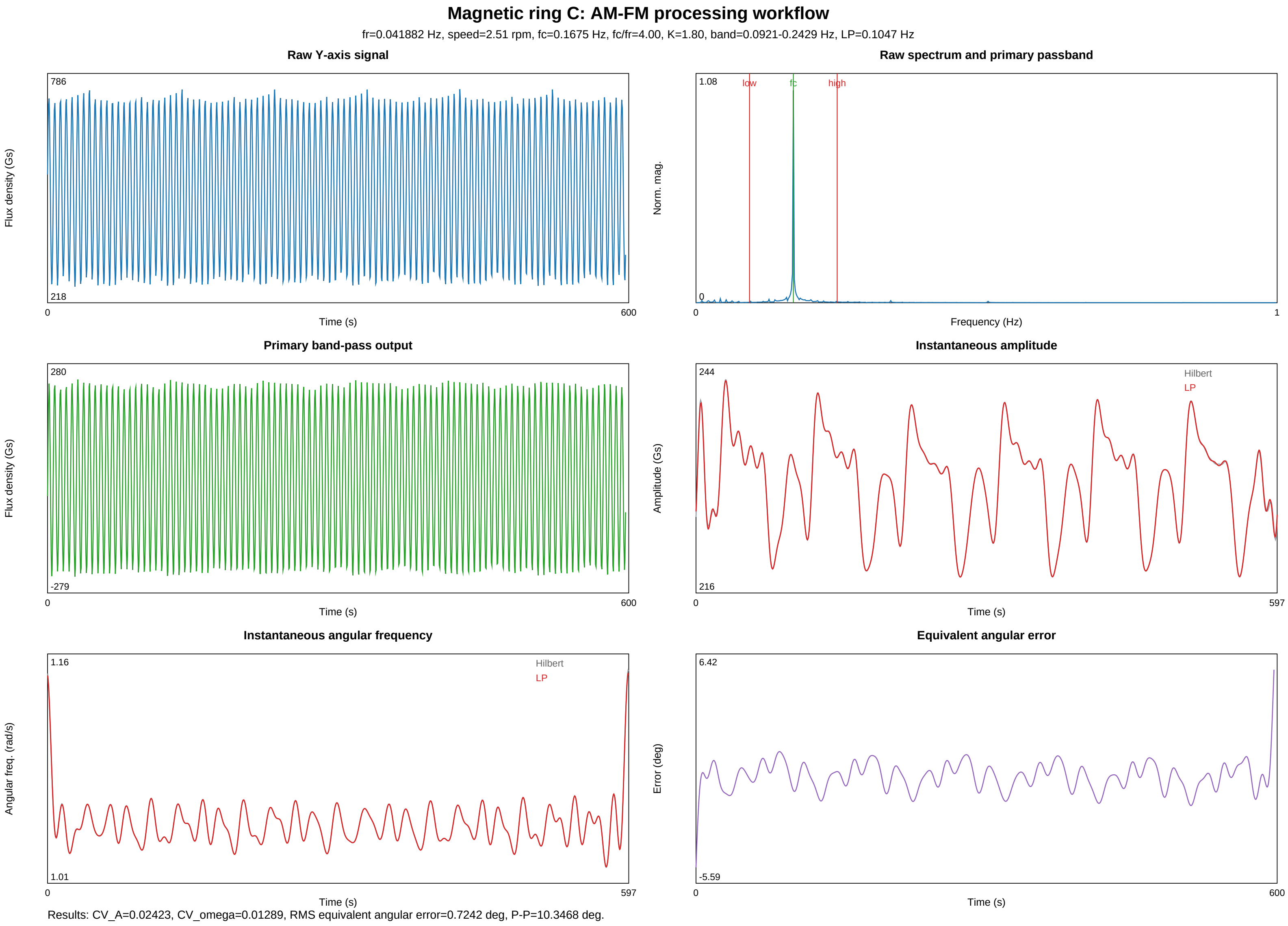

Figure S8: Processing details for magnetic ring C at $f_r = 0.042$ Hz.

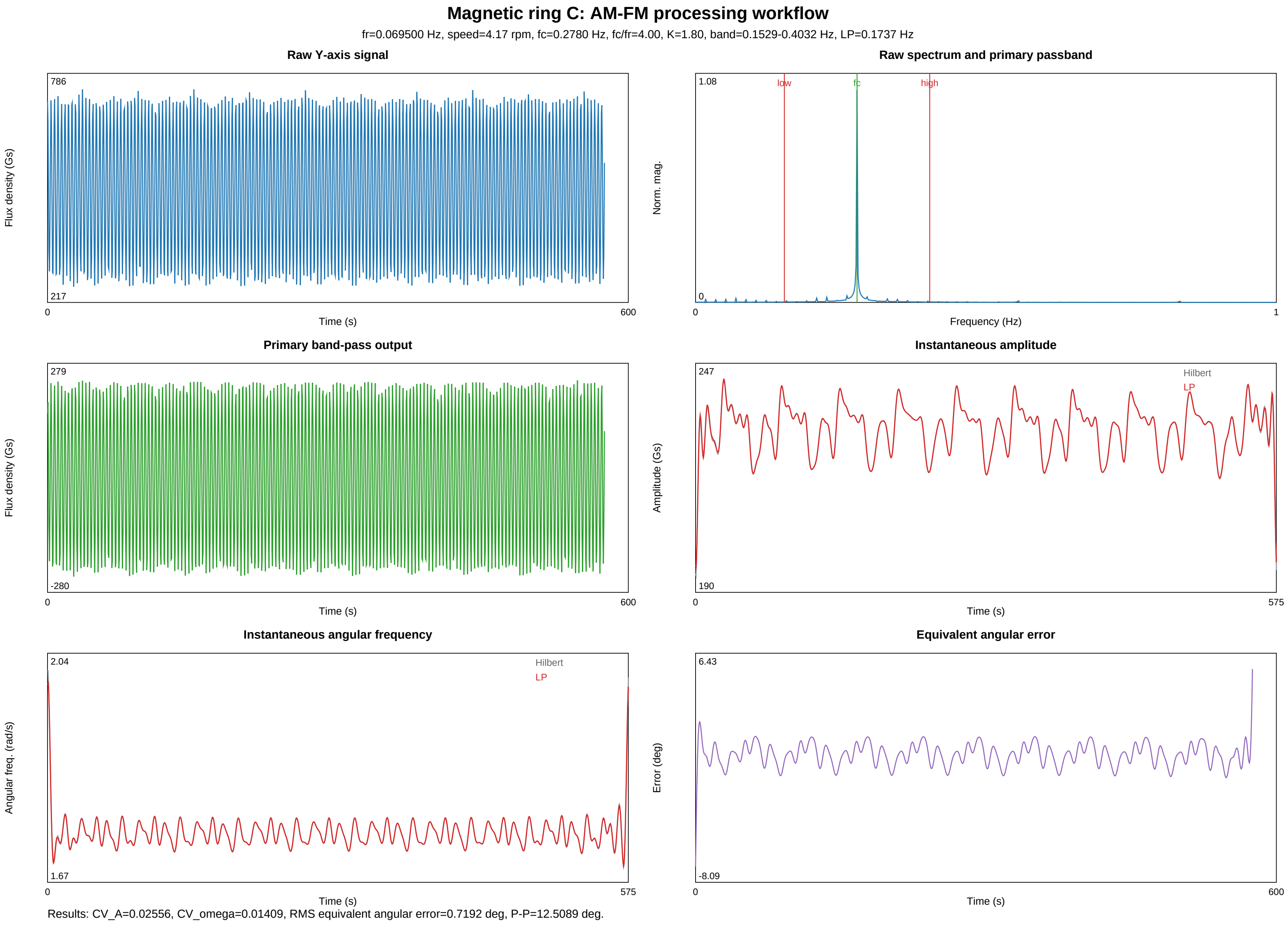

Figure S9: Processing details for magnetic ring C at $f_r = 0.069$ Hz.

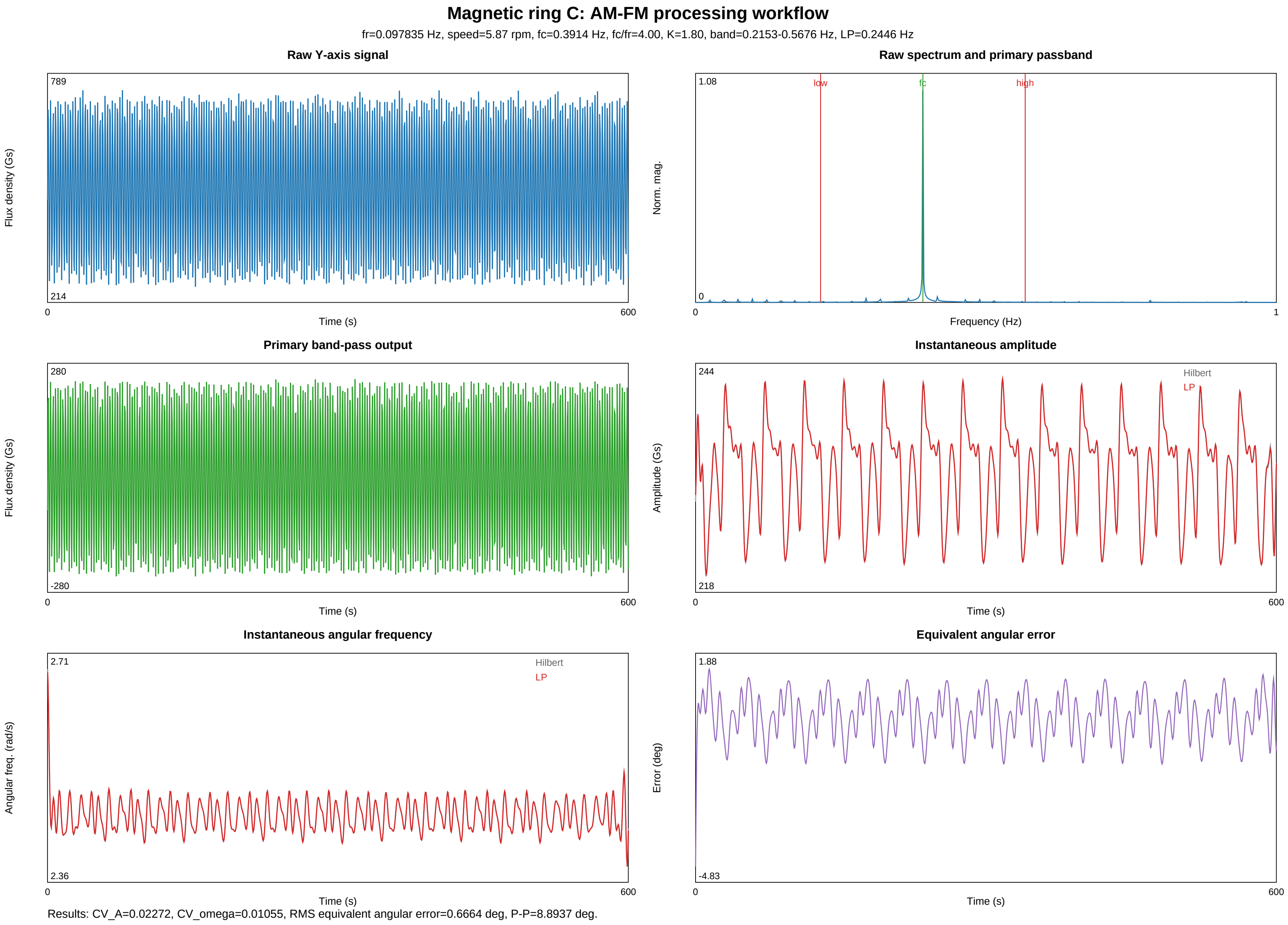

Figure S10: Processing details for magnetic ring C at $f_r = 0.098$ Hz.

S2. Selection of CV as the Relative-Fluctuation Descriptor

This section supports the use of CV-based indicators in the main text by comparing CV with common absolute fluctuation descriptors calculated from the demodulated instantaneous-amplitude sequence. Mean is the arithmetic average of the sequence, RMS is its root-mean-square level, standard deviation is the absolute dispersion around the mean, and P–P range is the difference between the maximum and minimum values. These four descriptors retain the physical unit of the source sequence, whereas CV is the ratio of standard deviation to mean and is dimensionless. Therefore, CV is more suitable for comparing relative fluctuation among states with different absolute signal levels.

For a processed sequence x_j $(j = 1, 2, \ldots, n)$, the candidate descriptors are calculated as

$$\begin{aligned}
&\text{Mean}: && \bar{x} = \tfrac{1}{n}\textstyle\sum_{j=1}^{n} x_j, \\
&\text{RMS}: && x_{\mathrm{RMS}} = \sqrt{\tfrac{1}{n}\textstyle\sum_{j=1}^{n} x_j^2}, \\
&\text{Standard deviation}: && \sigma_x = \sqrt{\tfrac{1}{n}\textstyle\sum_{j=1}^{n} (x_j - \bar{x})^2}, \\
&\text{P–P range}: && R_{\mathrm{P-P}} = x_{\max} - x_{\min}, \\
&\text{CV}: && \mathrm{CV}_x = \tfrac{\sigma_x}{\bar{x}}.
\end{aligned} \tag{S1}$$

Here, $x_{\max}$ and $x_{\min}$ are the maximum and minimum values of the processed sequence. In Table S2, x_j denotes the filtered instantaneous-amplitude sequence A_i.

Table S2: Candidate descriptors calculated from the demodulated instantaneous-amplitude sequence. Mean, RMS, standard deviation, and P–P range have the same unit as A_i, whereas CV is dimensionless.

Descriptor	Intact	Hidden crack	Visible 1 mm	Visible 2 mm	Trend	Interpretation for indicator selection
Mean	0.02479	0.21579	0.20349	0.16617	Non-monotonic	Strongly affected by absolute level and therefore unsuitable as the primary indicator.
RMS	0.02490	0.21752	0.21190	0.19322	Non-monotonic	Represents overall energy and still depends on amplitude scaling.
Standard deviation	0.00229	0.02736	0.05908	0.09859	Increasing	Sensitive to fluctuation intensity but retains physical scale.
P–P range	0.00892	0.10459	0.20179	0.31738	Increasing	Captures extreme variation but is more sensitive to local spikes.
CV	0.09247	0.12681	0.29034	0.59331	Increasing	Normalizes fluctuation by the mean and is therefore used as the primary descriptor of relative amplitude nonuniformity.

The same reasoning is used for the frequency-modulation descriptor CV_ω. Although the mean, RMS, standard deviation, and P–P range of ω_i would carry angular-frequency units, CV_ω is dimensionless and describes the relative fluctuation of instantaneous angular frequency. The paired use of CV_A and CV_ω therefore provides unit-independent descriptors for the AM and FM components, respectively.

S3. Effective Magnetic-Field Resolution and CV-Uncertainty Propagation

This section supports the precision discussion of the CV indicators in the main text by reporting the magnetic-field resolution of the measurement system and by giving the final dataset-specific standard uncertainties of the reported CV indicators. The visible-crack labels of 1 mm and 2 mm are nominal prepared severity states produced by filler-induced opening. They are not calibrated crack-width measurements and should be used only for qualitative severity ordering.

The analysis has two levels. First, the acquisition-chain readout resolution and the noise-limited effective magnetic-field resolution are evaluated from the DAQ voltage step, calibrated TMR voltage noise, and calibrated TMR sensitivity. Second, the CV indicators are obtained only after filtering, AM–FM demodulation, and normalization by the sequence mean. Therefore, the uncertainty of a CV result is not a fixed equipment constant and must be propagated for each processed dataset. The equipment and acquisition parameters used for the measurement-system resolution analysis are summarized in Table S3.

Table S3: Experimental equipment, acquisition settings, and calibration data used for the measurement-system resolution analysis.

Equipment or setting	Data used in processing	Value used in this study	Role in the resolution statement
MR5232 magnetic encoder ring	Number of magnetic poles	32 poles	Determines the pole-passage carrier and the number of pole periods within each complete revolution.
Zolix AK25-6520SR rotary stage	Set rotational speed and period	0.625 rpm; 96 s per revolution	Defines the complete-revolution segmentation used for comparable CV calculation.
DAQ6510 with 7700 module	Voltage acquisition range and readout increment	10 V range; 10^{-5} V readout increment	Defines the voltage readout step before conversion to magnetic flux density.
Acquisition program	Sampling interval and sampling rate	$\Delta t = 0.1$ s; 10 Hz	Determines the discrete sample spacing of $A_i[n]$ and $\omega_i[n]$.
Acquisition program	Raw and effective duration	750 s raw; 672 s after truncation	Gives seven complete revolutions for the crack-state comparison.
Acquisition program and 32-pole ring	Effective sample count	$672/0.1 = 6720$ samples; about 960 samples per revolution and 30 samples per pole	Defines the data length used to calculate the mean and standard deviation of each processed sequence.
Self-developed TMR read head with TMR2652D chip	Calibrated voltage noise and sensitivity	0.1611 mV RMS; 3.3350 mV/Gs	Provides the dominant noise-limited effective magnetic-field resolution before CV calculation.
TBR100 and TSA100-B positioning stages; HMC-C10 laser displacement sensor	Alignment and fixed lift-off control	Same installation and read-head position for the four crack states	Maintains comparable acquisition conditions; these controlled settings are not direct terms in the magnetic-field resolution calculation.

The voltage readout increment of the DAQ6510/7700 acquisition chain gives the acquisition-chain resolution. It is $\Delta V = 10^{-5}$ V on the 10 V range. Using the calibrated TMR sensitivity $S = 3.3350$ mV/Gs, the corresponding magnetic-field readout resolution is

$$\Delta B_{\mathrm{DAQ}} = \frac{10^{-5}\ \mathrm{V}}{3.3350 \times 10^{-3}\ \mathrm{V/Gs}} = 3.00 \times 10^{-3}\ \mathrm{Gs}. \tag{S2}$$

For comparison with RMS noise terms, if the quantization interval is represented by a rectangular distribution, the associated standard component is

$$u_{\mathrm{DAQ},B} = \frac{\Delta B_{\mathrm{DAQ}}}{\sqrt{12}} = 8.65 \times 10^{-4}\ \mathrm{Gs}. \tag{S3}$$

The calibrated TMR voltage-noise RMS is 0.1611 mV. With the same sensitivity, the equivalent noise-limited magnetic-field resolution is

$$u_{\mathrm{TMR},B} = \frac{0.1611\ \mathrm{mV}}{3.3350\ \mathrm{mV/Gs}} = 4.83 \times 10^{-2}\ \mathrm{Gs}. \tag{S4}$$

The effective magnetic-field resolution of the measurement system, expressed as an RMS noise-limited value, is therefore

$$u_B = \sqrt{u_{\mathrm{TMR},B}^2 + u_{\mathrm{DAQ},B}^2} = \sqrt{(4.83 \times 10^{-2})^2 + (8.65 \times 10^{-4})^2} = 4.83 \times 10^{-2}\ \mathrm{Gs}. \tag{S5}$$

This result shows that the system resolution is noise-limited by the calibrated TMR read head. The acquisition-chain magnetic-field readout resolution is approximately 3.00×10^{-3} Gs, whereas the effective noise-limited magnetic-field resolution is approximately 4.83×10^{-2} Gs RMS. Because the same read head, DAQ range, positioning procedure, and processing workflow were used for all crack states, the same measurement-system magnetic-field resolution applies to all four states.

For a processed sequence x_j containing n samples, x_j denotes either the filtered instantaneous-amplitude sequence $A_i[j]$ or the filtered instantaneous-angular-frequency sequence $\omega_i[j]$. The coefficient of variation is calculated from the mean and standard deviation as

$$\bar{x} = \frac{1}{n}\sum_{j=1}^{n} x_j, \qquad \sigma_x = \sqrt{\frac{1}{n}\sum_{j=1}^{n}(x_j - \bar{x})^2}, \qquad \mathrm{CV}_x = \frac{\sigma_x}{\bar{x}}. \tag{S6}$$

For the crack-state records, the raw duration of 750 s was truncated to seven complete revolutions. With a sampling interval of $\Delta t = 0.1$ s and an effective duration of 672 s, the processed sequence length is

$$n = \frac{672\ \mathrm{s}}{0.1\ \mathrm{s}} = 6720. \tag{S7}$$

Although the magnetic-field resolution above is common to the four measurements, the uncertainty of each reported CV value cannot be assigned from this common value alone. CV is a nonlinear relative descriptor because both the numerator σ_x and the denominator $\bar{x}$ are estimated from the processed sequence after band-pass filtering, Hilbert demodulation, low-pass filtering, and complete-revolution truncation. The propagation therefore depends on the local signal level, fluctuation amplitude, spectral content, filtering response, and sample count of each dataset. Therefore, the final CV uncertainty was evaluated at the processed-indicator level by applying the same AM–FM/CV workflow to analysis windows extracted from each crack-state record. Here, one window denotes one complete-revolution segment of the retained record; the same complete-revolution windowing was applied to both the amplitude- and frequency-related processed sequences. For a window-level indicator q_k ($k = 1, 2, \ldots, m$), where q_k denotes either CV_A or CV_ω calculated from the kth usable window, the window-level mean CV is

$$\bar{q} = \frac{1}{m}\sum_{k=1}^{m} q_k. \tag{S8}$$

For the visible 2 mm crack example listed below, the seven window-level CV_A values give $\bar{q} = 0.58438$. The final standard uncertainty was then evaluated from the repeatability of the window-level CV values as

$$s_{q,\mathrm{win}} = \sqrt{\frac{1}{m-1}\sum_{k=1}^{m}(q_k - \bar{q})^2}, \qquad u(q) = \frac{s_{q,\mathrm{win}}}{\sqrt{m}}. \tag{S9}$$

Here m is the number of usable analysis windows after applying the same truncation, filtering, demodulation, and transient-exclusion rules to the corresponding processed sequence. For both CV indicators, all four crack-state records were restricted to seven complete-revolution windows so that the same $m = 7$ was used for every state and for both indicators. The resulting uncertainty has the same dimensionless scale as the CV indicator. It is not a magnetic-field uncertainty in Gs; instead, it is the standard uncertainty of the final processed CV result. The final values are listed in Table S4.

Table S4: Final reported CV values and standard uncertainties of the crack-state indicators obtained after the complete AM–FM/CV workflow. The mean CV values are the reported main-text indicators, and the standard uncertainties are evaluated from seven complete-revolution windows and rounded to one significant digit.

Damage state	m	$\overline{\mathrm{CV}}_A$	$u(\mathrm{CV}_A)$	$\overline{\mathrm{CV}}_\omega$	$u(\mathrm{CV}_\omega)$
Intact	7	0.092	0.001	0.0044	0.0001
Hidden crack	7	0.127	0.005	0.0080	0.0002
Visible 1 mm crack	7	0.290	0.004	0.013	0.001
Visible 2 mm crack	7	0.593	0.004	0.04	0.01

Equivalently, the final values are reported as mean ± standard uncertainty in the main text: intact, $\mathrm{CV}_A = 0.092 \pm 0.001$ and $\mathrm{CV}_\omega = 0.0044 \pm 0.0001$; hidden crack, 0.127 ± 0.005 and 0.0080 ± 0.0002; visible 1 mm crack, 0.290 ± 0.004 and 0.013 ± 0.001; visible 2 mm crack, 0.593 ± 0.004 and 0.04 ± 0.01.

As an example, for the visible 2 mm crack state and the amplitude-related indicator, the seven complete-revolution window values were $q_k = 0.58383$, 0.57828, 0.59426, 0.56469, 0.59104, 0.58762, and 0.59096. The repeatability of these window-level CV values is first calculated as

$$s_{q,\mathrm{win}} = \sqrt{\frac{1}{7-1}\sum_{k=1}^{7}(q_k - \bar{q})^2} = 0.01017. \tag{S10}$$

The final standard uncertainty is then obtained from the standard error of the seven window-level values:

$$u(\mathrm{CV}_A) = \frac{s_{q,\mathrm{win}}}{\sqrt{m}} = \frac{0.01017}{\sqrt{7}} = 0.00384 \approx 0.004. \tag{S11}$$

This example shows the difference between the common magnetic-field resolution and the final CV uncertainty: the former characterizes the data-acquisition and TMR readout chain before signal processing, whereas the latter quantifies the repeatability of the dimensionless CV indicator after filtering, Hilbert demodulation, normalization, and window-level statistical evaluation.

S4. Disturbance-Separation Strategy by Speed Adjustment

This section supports the practical application discussion in Section 5.2 by showing that a known periodic disturbance can be separated from the useful carrier and crack-sensitive sidebands through rotational-speed adjustment. The present experiments do not independently vary temperature, strong structural vibration, or electromagnetic interference. These field-specific factors should be evaluated before deployment. The multi-speed results suggest that, when disturbance frequencies are known or measurable, the operating speed can be selected so that the pole-passage carrier and crack-sensitive low-order sidebands are separated from the disturbance components, allowing the carrier-centered band-pass filter and secondary low-pass filter to suppress them more effectively.

An additional disturbance-verification dataset was processed to illustrate this strategy. Datasets 1 and 2 were measured with the same imposed periodic disturbance but at different operating speeds, whereas dataset 3 was measured after removing the disturbance at a speed close to dataset 1. In dataset 1, the magnetic carrier remained close to the disturbance component near 0.249 Hz, so the carrier-centered passband partly overlapped the disturbance. In dataset 2, the operating speed was reduced and the magnetic carrier shifted to 0.0557 Hz; the same disturbance component was then outside the retained carrier band and could be rejected by the primary filtering step.

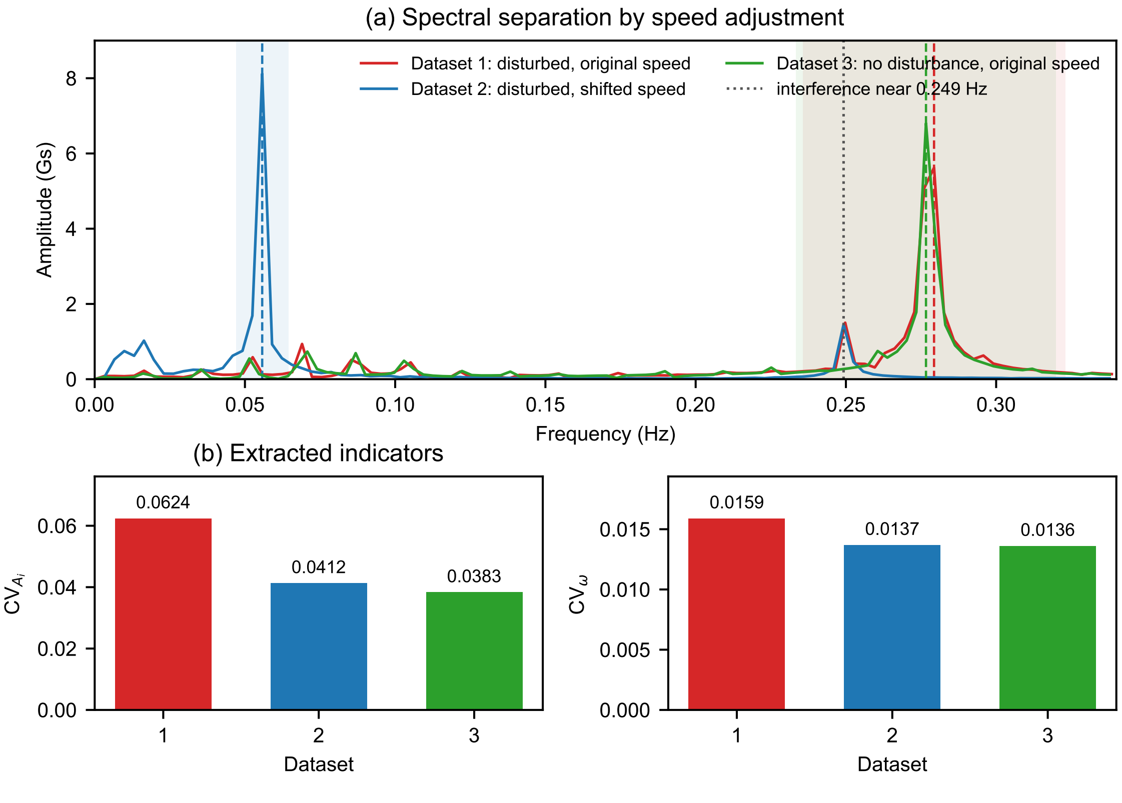

Figure S11: Disturbance suppression by speed adjustment. The spectral comparison shows that dataset 1 places the carrier band close to the imposed disturbance near 0.249 Hz, whereas dataset 2 shifts the useful carrier to a lower frequency band so that the same disturbance falls outside the retained passband. Dataset 3 is the no-disturbance reference at a speed close to dataset 1.

The shifted-speed result in dataset 2 is much closer to the no-disturbance reference in dataset 3 than to the disturbed original-speed result in dataset 1. The amplitude indicator decreases from

Table S5: Processed indicators for the disturbance-verification data.

Dataset	Condition	f_c (Hz)	Speed (rpm)	CV_A	CV_ω
1	Disturbance, original speed	0.2793	0.5237	0.06235	0.01589
2	Disturbance, shifted speed	0.0557	0.1045	0.04124	0.01368
3	No disturbance, original speed reference	0.2767	0.5187	0.03828	0.01360

0.06235 in dataset 1 to 0.04124 in dataset 2, approaching the no-disturbance value of 0.03828. The frequency-related indicator shows the same trend: $CV_\omega = 0.01368$ for dataset 2, which is almost the same as the no-disturbance reference value of 0.01360. These results support the practical interpretation that, when a periodic disturbance frequency is known, adjusting the rotational speed can move the useful magnetic carrier and crack-sensitive sidebands away from the disturbance, allowing the subsequent filtering and demodulation steps to suppress the interference more effectively.
